# Supplementary material for: Predictors of an early death in patients diagnosed with colon cancer: a retrospective case–control study in the UK
Source: BMJ Open. 2019 Jun 19;9(6):e026057. doi: 10.1136/bmjopen-2018-026057 (PMC6588982; doi:10.1136/bmjopen-2018-026057)
Supplement: Supplementary data [file bmjopen-2018-026057supp001.pdf]

Supplementary Table S1: Distribution of the case sample and the remaining patients who died within three months of diagnosis who were not included in the study

Characteristics		Case Sample n=484	Cases not included n=196	P value
Sex %	Male	52.7	44.3	
	Female	47.3	55.7	0.05[χ]
Year diagnosed %	2005-06	39.2	29.4	
	2007-08	38.5	18.40	
	2009-10	22.3	51.3	<0.01[χ]
Dukes stage %	A	2.3	1.0	
	B	10.1	7.0	
	C	10.7	9.0	
	D	37.0	44.3	
	Unknown	39.9	38.8	0.27[χ]
Deprivation quintile %	1 (least deprived)	16.6	15.2	
	2	20.9	22.7	
	3	17.4	18.7	
	4	24.8	21.7	
	5 (most deprived)	20.3	21.7	0.87[χ]
Mean age at diagnosis in years (Standard deviation)		76.5 (10.6)	77.6 (10.3)	0.83[†]
Mean survival duration in days (Standard deviation)		36.7 (24.7)	33.8 (39.9)	0.82[β]

P-values presented for chi squared tests (χ), t-tests (†) and Kaplan Meier (β)
