# Supplementary material for: Predictors of an early death in patients diagnosed with colon cancer: a retrospective case–control study in the UK
Source: BMJ Open. 2019 Jun 19;9(6):e026057. doi: 10.1136/bmjopen-2018-026057 (PMC6588982; doi:10.1136/bmjopen-2018-026057)
Supplement: Author's manuscript [file bmjopen-2018-026057.draft_revisions.pdf]

BMJ Open

BMJ Open is committed to open peer review. As part of this commitment we make the peer review history of every article we publish publicly available.

When an article is published we post the peer reviewers' comments and the authors' responses online. We also post the versions of the paper that were used during peer review. These are the versions that the peer review comments apply to.

The versions of the paper that follow are the versions that were submitted during the peer review process. They are not the versions of record or the final published versions. They should not be cited or distributed as the published version of this manuscript.

BMJ Open is an open access journal and the full, final, typeset and author-corrected version of record of the manuscript is available on our site with no access controls, subscription charges or pay-per-view fees (http://bmjopen.bmj.com).

If you have any questions on BMJ Open's open peer review process please email info.bmjopen@bmj.com

BMJ Open

Predictors of an early death in patients diagnosed with colon cancer: a retrospective case-control study in the United Kingdom

Journal:	*BMJ Open*
Manuscript ID	bmjopen-2018-026057
Article Type:	Research
Date Submitted by the Author:	31-Aug-2018

Complete List of Authors:	Donnelly, Conan; National Cancer Registry Ireland, University of Cork<br>Hart, Nigel; Queen's University Belfast, School of Medicine, Dentistry and Biomedical Sciences - Centre for Public Health<br>McCrorie, Alan; Queen's University Belfast, Centre for Cancer Research and Cell Biology<br>Donnelly, Michael; Queen's University Belfast, Centre for Public Health<br>Anderson, Lesley; Queen's University Belfast, School of Medicine<br>Ranaghan, Lisa; Belfast Health and Social Care Trust<br>Gavin, Anna; Queen's University Belfast, N. Ireland Cancer Registry

Keywords:	Epidemiology < ONCOLOGY, STATISTICS & RESEARCH METHODS, Gastrointestinal tumours < ONCOLOGY, Colorectal surgery < SURGERY

SCHOLARONE™
Manuscripts

Conan Donnelly[1*], Nigel Hart[2], Alan D McCrorie[3] Michael Donnelly[4], Lesley A Anderson[4], Lisa Ranaghan[5], Anna Gavin[1]

1. N. Ireland Cancer Registry, Queen's University Belfast, 2. Department of General Practice, Queen's University Belfast, 3. School of Medicine, Dentistry and Biomedical Science, Queen's University Belfast. 4. Centre for Public Health, Queen's University Belfast. 5. Belfast, Health and Social Care Trust.

*Corresponding author: c.donnelly@nicr.ie, National Cancer Registry Ireland, Cork Airport Business Park, Cork, T12 CDF7

TITLE: Predictors of an early death in patients diagnosed with colon cancer: a retrospective case-control study in the United Kingdom

(Running title: Predictors of an early death from colon cancer)

KEY WORDS

Colon Cancer, Survival, health seeking, early death, early diagnosis

FUNDING

The TEDI study was funded by the Cancer Research UK led National Awareness & Early Diagnosis Initiative (Principal Investigator: AG, Co-Investigators: CD, NH, AM, MD, LR, LA). The Northern Ireland Cancer Registry is funded by the Public Health Agency Northern Ireland (Principal Investigator: AG)

COMPETING INTERESTS

There are no competing interests to be reported by the authors in this study

WORD COUNT: 3923

FIGURES: 1, TABLES: 5, SUPPLEMENTARY TABLES: 1

For peer review only - http://bmjopen.bmj.com/site/about/guidelines.xhtml

ABSTRACT

OBJECTIVE: Despite considerable improvements, five-year survival rates for colon cancer in the United Kingdom (UK) remain poor when compared with other socioeconomically similar countries. Variation in five-year survival can be partly explained by higher rates of death within three months of diagnosis in the UK. This study investigated characteristics of patients who died within three months of a diagnosis of colon cancer with the aim of identifying specific patient factors that can be addressed or accounted for to improve survival outcomes.

DESIGN: A retrospective case-control study design was applied with matching on age, sex and year diagnosed. Colon cancer patient, disease, clinical and service characteristics, diagnosed in a UK region (2005-2010), who survived less than three months from diagnosis (cases) were compared with patients who survived between six and thirty-six months (controls). Patient and clinical data was sourced from General Practice notes and hospital databases 1-3 years pre-diagnosis.

RESULTS: Being older (aged ≥78 years) and living in deprivation quintile 5 (OR=2.64, CI: 1.15-6.06), being unmarried and living alone (OR=1.64, CI: 1.07-2.50), being underweight compared to normal weight or obese (OR=3.99, CI: 1.14-14.0) and being older and living in a rural as opposed to urban area (OR=1.96, CI: 1.21-3.17) were all independent predictors of early death from colon cancer. Missing information was also associated with early death including unknown stage, histological type and marital/accommodation status after accounting for other factors.

CONCLUSION: Several factors typically associated with social isolation were a recurring theme in patients who died early from colon cancer death. This association is unexplained by clinical or diagnostic pathway characteristics. Socially isolated patients are a key target group to improve outcomes of the worst surviving patients but further investigation is required to determine if being isolated itself is actually a cause of early death from colon cancer.

For peer review only

Strengths and limitations of this study

- Study sample was generated from a high-quality population-based cancer registry system with relatively few death certificate only (DCO) cases.
- Case-control design provided an efficient method of collecting data and allowed development of a control group that was matched on important non-modifiable characteristics.
- Survival of controls was restricted to a population of patients whose survival was less than three years and similar to the case population.
- Study identified several characteristics which discriminated between cases and controls suggesting that patients who die within the first few months of diagnosis are a specific patient cohort who require attention.

For peer review only

For peer review only - http://bmjopen.bmj.com/site/about/guidelines.xhtml

INTRODUCTION

Despite considerable improvements, United Kingdom (UK) survival rates for colon cancer remain poor by international comparison with higher five-year survival reported in Norway, Sweden, Canada and Australia[1] and poorer survival in the UK compared to several countries reported in Eurocare[2]. These deficits have largely been explained by survival at three months post diagnosis[3]. Patients who survive beyond this period in the UK have similar five-year survival rates to their counterparts in better performing countries[1]. Approximately 19% of colorectal cancer patients in the UK and 16% in the Northern Ireland (NI) died within three months of diagnosis between 2006 and 2008[4, 5]. It was estimated that if survival in England matched that of Norway, 13.6% fewer patients would die within the three month period[3]. Generally poor survival is linked with a number of factors including late stage disease at diagnosis[6], poor patient fitness due to coexisting disease[7] and limited availability of and access to high quality investigations and treatment[8].

Reasons for diagnostic delay in colorectal cancer are well documented[9]. Lower educational status[10, 11] and rural residence[12, 13] have been associated with delayed help seeking. Additionally, stronger social networks have been associated with shorter diagnostic delay[14, 15, 16]. Clinical characteristics also play a role, patients with co-morbid disease[11, 17] and/or multiple symptoms[11] are less likely to delay compared to those with non-specific symptoms[17, 18]. Application of referral guidelines by General Practitioners (GPs) has been shown to reduce delay[19] while younger patients[13, 20], those of lower socio-economic status[21] and frequent help seekers[10, 16] were less likely to be referred. While bowel cancer screening was introduced in the UK in 2007[22] and in NI in 2011[23], the vast majority patients are diagnosed clinically[24], therefore the role of clinical decision making in early colon cancer diagnosis remains paramount.

The relationship between these factors and surviving past the first few months following a colon cancer diagnosis has not been adequately investigated and their role in explaining

For peer review only - http://bmjopen.bmj.com/site/about/guidelines.xhtml

international survival differences requires attention. The aim of this study was to investigate patient, clinical and disease factors associated with early death in colon cancer patients in Northern Ireland and to determine factors which might help to identify subgroups in the population for early diagnosis interventions.

METHODOLOGY

This study employed a retrospective, individually matched, case-control design involving a posthumous review of primary care physician or GP and electronic secondary care notes. The study design was guided by the principles of the Aarhus statement on early diagnosis research[25]. Principles adhered to in this study include items 1-4, 7-9, and 20 of the Aarhus checklist. Date of initial cancer diagnosis is defined by the NICR as date of first tissue diagnosis in secondary care, not as symptom presentation in primary and/or secondary care.

Case and control definition and identification

Cases

Patients diagnosed with primary colon cancer (ICD 10: C18) in NI between January 2005 and December 2010 were identified using the Northern Ireland Cancer Registry (NICR). Using death registrations, provided by the NI General Registrar Office, the status and survival of patients was determined. Cases were defined as patients with an observed survival of under 90 days following diagnosis date (as assigned by NICR). A random sample of all eligible cases was selected using random number tables based on pre-defined power calculations.

Controls

Controls were patients with an observed survival lasting over six months and less than three years leaving a three month buffer between the survival rates of cases and controls. Controls were individually matched, using individual nearest neighbour matching[26], [27], to cases by age (within 5-year age bands), sex and year of diagnosis (within 2-year groups).

For peer review only - http://bmjopen.bmj.com/site/about/guidelines.xhtml

In both groups, patients with incident cancer identified by death certificate only (DCO) and patients with recurrence of a previous incident colon cancer were excluded.

Exposure variables and covariates

Data items were identified through literature review with items and categorisation defined in consultation with a clinical adviser, GP, a colorectal surgeon and an oncologist. Items were classified into seven areas: demographic [marital status, accommodation status, NI Multiple Deprivation Measure (NI MDM quintile), rural/urban status], lifestyle (smoking and alcohol status, health seeking activity including uptake of flu vaccine and frequency of GP attendance) and co-morbidities (Charlson Co-Morbidity score [CCI] and psychiatric illnesses). These characteristics were collected from information recorded between one and three years before diagnosis. Marital and accommodation status were merged in final analysis due to multicollinearity. Disease characteristics included symptoms in the year to diagnosis, disease stage at diagnosis with histology, morphology and grade collected from pathology records held in the NICR. GP and hospital episodes [including symptoms (classified as 'vague' or 'alarm' based on NICE Guidelines for Suspected Cancer Referral guidelines), clinician actions (number of GP episodes before diagnosis and referral) and investigations ordered]. In addition, treatment [first treatment type, treatment intent, surgical resection (y/n), radiotherapy (y/n), chemotherapy (y/n)] and death information (date, place and cause of death) were also collected. Data were collected by two trained data abstractors under the guidance of a medically trained clinical adviser using a common bespoke proforma. Data was sourced from GP records, electronic hospital records including the hospital discharge records, multidisciplinary team (MDT) and oncology data systems. The study target sample was 600 cases matched to 600 controls with this sample it was estimated the study would have over 90% power to detect (at the 5% level) a 10-percentage point difference in the proportion of married cases vs controls where 47% of the control group were married.

For peer review only - http://bmjopen.bmj.com/site/about/guidelines.xhtml

Statistical method

Data was analysed using 'STATA 14 (StataCorp 2015)'. All missing data were categorised as unknown and included in the analysis. Univariate analysis involved cross-tabulation of all categorical variables with case/control status. Conditional logistic regression (CLR) was used to produce unadjusted odds ratios and associated 95% confidence intervals to identify independent factors associated with early death. Patient characteristics, significant at the $p<0.25$, were included in a minimally adjusted multivariable model to test independence from other co-morbidities, patient and disease characteristics. Stage and pathway to diagnosis characteristics (number of A&E and GP episodes in three months preceding diagnosis) were added to the models to assess the degree to which they explained variation in early death among different patient groups. Age (a binary classification around median age [78 years] of cases) and sex stratified univariate and multivariable analysis were undertaken to investigate differences in patterns in early death between these groups.

Patient and public involvement

Members of the public including patients were not involved in the design or analysis stages of this piece of non-interventional research but research question was designed to explore characteristics of patients who die early after a diagnosis of colon cancer. Ethical approval for this study was granted by the Office of Research Ethics Committees Northern Ireland (12/NI/0034). This committee receives input from lay member(s) of the public before reaching a decision about whether or not to approve research studies.

RESULTS

There were 4,358 colon cancer tumours between 2005 and 2010 registered by the NICR. Of these, 743 (17%) related to patients who died within 3 months of diagnosis and 1,069 related to patients who died between six months and three years. Following exclusions and sampling (Figure 1), 484 cases and the same number of matched controls were generated.

For peer review only - http://bmjopen.bmj.com/site/about/guidelines.xhtml

There were no significant differences between cases included in the study and those not included (Supplementary Table 1) regarding stage at diagnosis, deprivation quintile, age and survival. However, the study group included significantly more males than females as well as fewer patients diagnosed in 2009 and 2010 due to resource constraints in data collection.

Univariate analysis

Compared to married patients, odds of early death were higher among single, widowed and those with unknown marital status (Table 1). Those who lived alone, in nursing or residential care or were living with another relative were more likely to die within three months compared to those living with a spouse/partner. Odds of early death were also higher in the most deprived communities (23%) compared to the least deprived (13%) (Table 1).

Table 1: Demographic characteristics of cases and controls and associated odds ratios for early death and 95% confidence intervals

Characteristic		Case		Control		OR†	95% CI
		n	%	n	%		
Accommodation status	Spouse/ partner	156	32.2	233	48.1	1	
	Nursing/residential	48	9.9	23	4.8	3.93	2.18 – 7.09
	Sheltered dwelling	8	1.7	14	2.9	0.91	0.37 – 2.24
	Alone	156	32.2	152	31.4	1.74	1.23 – 2.45
	Lives with relative	53	11.0	40	8.3	2.32	1.41 – 3.82
	Unknown	63	13.0	22	4.6	5.32	3.00 – 9.43
Marital status	Married/Cohabiting	189	39.1	257	53.1	1	
	Divorced	15	3.1	13	2.7	1.51	0.66 – 3.45
	Unknown	44	9.1	15	3.1	3.79	2.06 – 6.96
	Single	74	15.3	53	11.0	1.88	1.25 – 2.84
	Widowed	162	33.5	146	30.2	1.60	1.14 – 2.22

For peer review only - http://bmjopen.bmj.com/site/about/guidelines.xhtml

Urban/rural status	Rural	178	63.2	166	65.7	1	
	Urban	306	36.8	318	34.3	0.89	0.68 – 1.17
Deprivation quintile	Q1 (least deprived)	64	13.2	89	18.4	1	
	Q2	95	19.6	92	19.0	1.45	0.76 – 1.74
	Q3	106	21.9	111	22.9	1.01	0.66 – 1.54
	Q4	110	22.7	102	21.1	1.49	0.99 – 2.24
	Q5 (most deprived)	109	22.5	90	18.6	1.47	0.95 - 2.27
Flu uptake	No uptake	71	14.7	47	9.7	1	
	≥1 vaccination	324	66.9	345	71.3	0.63	0.43-0.94
	Unknown	89	18.4	92	19.0	0.65	0.40-1.05
Baseline consultation activity (tertile)	<11	149	30.8	156	32.2	1	
	11-19	148	30.6	162	33.5	0.98	0.71-1.34
	>=20	187	38.6	166	34.3	1.20	0.87-1.67
Smoking	Non-smoker	221	45.7	219	15.7	1	
	Ex-smoker	141	29.1	175	36.2	0.76	0.55 – 1.04
	Current smoker	91	18.8	76	15.7	1.19	0.81 – 1.75
	Unknown	31	6.4	14	2.9	2.30	1.16 – 4.56
Alcohol Consumption	Current drinker	154	31.8	175	36.2	1	
	Ex-drinker	31	6.4	26	5.4	1.37	0.78 – 2.43
	Never drank	189	39.1	191	39.5	1.13	0.82 – 1.55
	Unknown	110	22.7	92	19.0	1.38	0.96 – 1.99

† Conditional logistic regression odds ratios for cases and controls matched on age, sex and year diagnosed

For peer review only

Baseline GP consultation activity was not associated with early death. Regarding flu vaccine uptake, it was not possible to identify patients who were invited for flu vaccine though based on age alone, 86% of cases were eligible. Approximately 70% took the flu vaccine at least once during the 1-3 years period before diagnosis; 15% did not attend for the flu vaccine and attendance for the remaining 18% was unknown. Patients who attended twice in the 1-3 year

For peer review only - http://bmjopen.bmj.com/site/about/guidelines.xhtml

period before diagnosis had lower odds of early death than patients who did not attend. Smoking status was not significantly associated with early death with the exception of those who had an unknown smoking status (see Table 1).

Being underweight (BMI <18.5) was strongly associated with early death compared to patients with a normal BMI. Furthermore, obesity was not associated with early death (Table 2). Co-morbidity was common among patients who died early. Almost three quarters (72%) had at least one co-morbidity. CCI score was, however, not associated with early death (mean CCI score for cases was 4.75 compared to 4.90 for controls (p=0.32). Dementia was the only co-morbidity within the CCI that was associated with early death - it was present in 8% of cases compared to 4% of controls (Table 2).

Table 2: Presence of individual co-morbidities included in the Charlson score in cases and controls and associated clogit odds ratios and 95% confidence intervals

Characteristic		Case		Control		OR	95% CI
		n	%	n	%		
Underweight	No	467	96.5	479	99.0	1	
	Yes	17	3.5	5	1.0	3.4	1.25–9.22
Obese	No	425	87.8	420	86.8	1	
	Yes	59	12.2	64	13.2	0.91	0.63–1.33
Dementia	No	445	91.9	462	95.5	1	
	Yes	39	8.1	22	4.6	1.85	1.07–3.19
Hypertension	No	279	57.6	260	53.7	1	
	Yes	205	42.4	224	46.3	0.84	0.6–1.10
Ischaemic heart disease	No	391	80.8	386	79.8	1	
	Yes	93	19.2	98	20.3	0.94	0.68–1.29
Parkinson's Disease	No	473	97.7	477	98.6	1	
	Yes	11	2.3	7	1.5	1.57	0.61–4.05
Valvular heart	No	422	87.2	416	86.0	1	

For peer review only - http://bmjopen.bmj.com/site/about/guidelines.xhtml

disease	Yes	62	12.8	68	14.1	0.89	0.61–1.31
Myocardial Infarction	No	429	88.6	436	90.1	1	
	Yes	55	11.4	48	9.9	1.16	0.78–1.72
Congestive heart failure	No	459	94.8	451	93.2	1	
	Yes	25	5.2	33	6.8	0.72	0.41–1.27
Peripheral vascular disease	No	452	93.4	462	95.5	1	
	Yes	32	6.6	22	4.6	1.53	0.86–2.72
Cerebrovascular disease	No	433	89.5	440	90.9	1	
	Yes	51	10.5	44	9.1	1.18	0.77–1.81
COPD	No	402	83.1	397	82.0	1	
	Yes	82	16.9	87	18.0	0.98	0.64–1.50
Connective tissue disorder	No	441	91.1	440	90.9	1	
	Yes	43	8.9	44	9.1	0.98	0.64–1.50
Diabetes without complications	No	420	86.8	429	88.6	1	
	Yes	64	13.2	55	11.4	1.20	0.81–1.77
Peptic ulcer	No	446	92.2	456	94.2	1	
	Yes	38	7.9	28	5.8	1.40	0.84–2.34
Liver disease	No	481	99.4	481	99.4	1	
	Yes	3	0.6	3	0.6	1	0.20–4.95
Hemiplegia / Paraplegia	No	484	100.0	482	99.6	1	
	Yes	0	0.0	2	0.4	-	-
Renal disease	No	441	91.1	449	92.8	1	
	Yes	43	8.9	35	7.2	1.24	0.79–1.94
Diabetes with complications	No	465	96.1	460	95.1	1	
	Yes	19	3.9	24	5.0	0.79	0.43–1.45
Cancer	No	468	96.7	456	94.2	1	
	Yes	16	3.3	28	5.8	0.57	0.31–1.06
Leukaemia	No	482	99.6	482	99.6	1	

For peer review only

For peer review only - http://bmjopen.bmj.com/site/about/guidelines.xhtml

	Yes	2	0.4	2	0.4	1.00	0.14–7.10
Lymphoma	No	482	99.6	483	99.8	1	
	Yes	2	0.4	1	0.2	2.00	0.18–22.06
Severe liver disease	No	484	100	483	99.8	1	
	Yes	0	0	1	0.2	-	-
Metastatic cancer	No	451	93.2	436	90.0	1	
	Yes	33	6.8	48	9.9	0.64	0.40–1.04

†Conditional logistic regression odds ratios for cases and controls matched on age, sex and year diagnosis

Regarding pre-existing psychiatric conditions, 1% of cases were recorded as having schizophrenia, 1% with a learning disability and 13% with anxiety or depression. None were significantly associated with early death. However, the small number of patients with ‘other’ psychiatric conditions had higher odds of early death (Table 3). Compared to Dukes Stage A, Dukes Stage D and unknown stage were associated with early death. Unknown histological type, unspecified anatomical site and undetermined grade were also associated with early death. Patients with a family history of colorectal cancer had lower odds of early death compared to patients without a family history (Table 4).

Table 3: Psychiatric illness among cases and controls and associated clogit odds ratios and 95% confidence intervals

Characteristic		Case		Control		OR	95% CI
		n	%	n	%		
Learning disability	No	479	99.0	482	99.6	1	
	Yes	5	1.0	2	0.4	1.24	0.85–1.79
Anxiety/Depression	No	419	86.6	425	87.8	1	
	Yes	65	13.4	59	12.2	1.13	0.76–1.70
Schizophrenia	No	480	99.2	479	99.0	1	
	Yes	4	0.8	5	1.0	0.80	0.22–2.98
Other psychiatric	No	470	97.1	480	99.2	1	

For peer review only - http://bmjopen.bmj.com/site/about/guidelines.xhtml
For peer review only

disorder	Yes	14	2.9	4	0.8	3.50	1.15–10.63

†Conditional logistic regression odds ratios for cases and controls matched on age, sex and year diagnosis

Table 4: Disease characteristics among cases and controls and associated odds ratios and 95% confidence intervals

**Characteristic**		**Case**		**Control**			
		**n**	**%**	**n**	**%**	**OR†**	**95% CI**
Anatomical Location	Ascending	52	10.7	57	11.8	1	
	Caecum	93	19.2	136	28.1	0.83	0.52–1.29
	Other	90	18.6	93	19.2	1.19	0.73-1.93
	Descending	29	6.0	18	3.7	1.97	0.97–3.99
	Sigmoid colon	95	19.6	132	27.3	0.86	0.54–1.37
	Not specified	125	25.8	48	9.9	2.96	1.75-5.02
Histological type	Adenocarcinoma	256	52.9	384	79.3	1	
	Mucinous	14	2.9	28	5.8	0.91	0.45–1.81
	Not specified	198	40.9	60	12.4	5.62	3.80–8.34
	Other	16	3.3	12	2.5	1.68	0.77–3.67
Metastases	None	37	7.6	64	13.2	1	
	Bone	9	1.9	1	0.2	17.2	2.48-143.6
	Liver	134	27.7	108	22.3	2.37	1.42-3.95
	Lung	14	2.9	10	2.1	2.55	1.01-6.38
	Other	27	5.6	23	4.8	2.17	1.07-4.41
	Unknown	263	54.3	278	57.4	1.77	1.10-2.84
Dukes Stage	A	8	1.7	19	3.9	1	
	B	46	9.5	85	17.6	1.37	0.54–3.48
	C	47	9.7	127	26.2	1.02	0.41–2.52

For peer review only - http://bmjopen.bmj.com/site/about/guidelines.xhtml

	D	160	33.1	140	28.9	2.96	1.23–7.14
	Unknown	233	46.1	113	23.4	5.65	2.30–13.91
Grade (differentiation)	Well/Moderate	93	19.2	190	39.3	1	
	Poor/ Undifferentiated	40	8.3	57	11.8	1.45	0.89-2.36
	Not determined	351	72.5	237	50.0	3.32	2.38-4.62
Colorectal polyps	No	467	10.7	57	11.8	1	
	Yes	17	19.2	136	28.1	0.80	0.42–1.54
Bowel cancer family history	No	459	25.8	48	9.9	1	
	Yes	25	6.0	18	3.7	0.52	0.31–0.88

†Conditional logistic regression odds ratios for cases and controls matched on age, sex and year diagnosis

Multivariable analysis

Unknown marital status, being single, widowed, divorced and living alone were all associated with early death compared to patients who were married/co-habiting after adjusting for other patient characteristics and co-morbidities. Regarding socio-economic status, a deprivation gradient for early death was apparent in older people living within quintile 5. This relationship existed for all patients in quintile 4. These socially deprived groups had higher odds of early death compared to the least deprived after adjusting for other factors (Table 5). Being under-weight between 1-3 years before diagnosis was significantly associated with early death in multivariable analysis. Unspecified histology and Dukes Stage D disease remained positively associated with early death in multivariable analysis.

Table 5: Multivariable analysis of the association between patient characteristics and early death

	All patients			Male			Female			Aged ≥78 year			Aged <78 year		
	**$n^{\neq}$**	**OR**	**95 %**	**$n^{\neq}$**	**OR**	**95 %**	**$n^{\neq}$**	**OR**	**95 %**	**$n^{\neq}$ α**	**OR**	**95 %**	**$n^{\neq}$ α**	**OR**	**95 %**

For peer review only - http://bmjopen.bmj.com/site/about/guidelines.xhtml

				CI			CI			CI			CI			CI
Marital / accommodation status	Married/co-habiting	371	1.00		248	1.00		123	1.00		125	1.00		246	1.00	
	Married/not co-habiting	26	1.90	0.65-5.57	11	1.27	0.27-6.01	15	3.72	0.70-19.7	17	1.60	0.45-5.67	9	0.63	0.04-10.1
	Institutional care	63	1.93	0.93-4.00	25	1.78	0.57-5.57	38	2.60	0.85-7.82	52	3.02	1.22-7.49	11	0.15	0.01-1.48
	Unknown	127	5.01	2.82-8.89	66	3.87	1.76-8.38	61	8.49	3.12-23.1	68	4.32	1.97-9.47	59	6.36	2.08-19.5
	Living alone & SWD*	270	1.64	1.07-2.50	113	1.55	0.86-2.80	157	2.21	1.01-4.80	168	1.62	0.90-2.91	102	1.52	0.72-3.21
	Other & SWD*	111	1.39	0.80-2.44	47	1.28	0.59-2.74	64	2.03	0.76-5.44	61	0.85	0.37-1.95	50	2.55	0.96-6.82
Deprivation quintile	Q1 Least deprived	153	1.00		87	1.00		66	1.00		83	1.00		70	1.00	
	Q2	187	1.28	0.74-2.25	91	0.84	0.39-1.82	96	2.90	1.10-7.6	96	2.29	1.06-4.93	91	0.60	0.21-1.74
	Q3	217	1.42	0.83-2.42	112	1.28	0.62-2.63	105	2.09	0.81-5.4	107	2.05	0.96-4.38	110	1.09	0.41-2.93
	Q4	212	1.88	1.09-3.23	112	1.60	0.79-3.22	100	3.10	1.14-8.4	113	2.02	0.98-4.15	99	1.71	0.62-4.67

For peer review only

	Q5 Most deprived	199	1.72	0.98-3.02	108	1.13	0.53-2.33	91	4.70	1.68-13.2	92	2.64	1.15-6.06	107	1.17	0.43-3.16
Being underweight (BMI <18.5)		22	3.99	1.14-14.0	6	2.74	0.32-21.1	16	3.96	0.65-24.2	13	7.27	1.42-68.8	9	2.34	0.24-22.6
Dukes stage	A	8	1.00		20	1.00		7	1.00		10	1.00		17	1.00	
	B	46	1.85	0.64-5.32	70	1.72	0.52-5.67	61	4.90	0.32-75.1	69	2.43	0.43-13.7	62	1.08	0.24-4.06
	C	47	1.51	0.54-4.27	82	1.13	0.33-3.82	92	6.04	0.43-83.9	88	2.17	0.41-11.6	86	0.87	0.18-4.28
	D	160	3.07	1.13-8.38	162	1.98	0.63-6.21	138	13.8	1.02-188	109	4.64	0.84-25.7	191	1.86	0.44-7.84
	Unknown	233	2.99	1.05-8.53	176	1.86	0.58-6.02	160	15.8	1.06-236	215	4.27	0.79-22.9	121	1.72	0.34-8.78
Histology type	Adenocarcinoma	440	1.00		352	1.00		288	1.00		296	1.00		344	1.00	
	Mucinous	42	1.20	0.54-2.66	17	2.13	0.59-7.64	25	0.87	0.27-2.72	22	1.06	0.35-3.27	20	0.98	0.24-4.06
	Not specified	258	3.66	2.17-6.17	126	5.02	2.47-10.2	132	2.01	0.82-4.90	166	2.70	1.37-5.31	92	10.9	3.13-38.0
	Other	28	1.16	0.48-2.83	15	1.58	0.44-5.66	13	0.76	0.17-3.46	7	0.58	0.09-3.77	21	1.49	0.45-4.95

For peer review only

For peer review only - http://bmjopen.bmj.com/site/about/guidelines.xhtml

Rural residence	344	1.40	0.98-2.01	182	1.29	0.78-2.17	162	1.53	0.86-2.74	178	1.96	1.21-3.17	166	0.76	0.38-1.52

**=SWD= single, widowed, divorced*

≠: n= cases and controls combined

Additional models that adjusted for pathway characteristics (attendance at A&E and number of GP consultations prior to diagnosis) did not explain the association between marital status and early death ($p<0.01$). Flu vaccination attendance and baseline consultation activity, dementia, psychiatric illness and smoking status were not significantly associated with early death in multivariable analysis.

Consistent patterns were observed among males and females when data was stratified by sex though the association between deprivation and early death was strongly pronounced among women and not present among men. Age stratification showed the odds of early death were higher for those living in rural areas compared to urban areas among patients aged >=78 years. This association was not apparent in the younger age group. With this exception, there were only minor differences in the factors associated with early death between the two age groups (see Table 5).

For peer review only - http://bmjopen.bmj.com/site/about/guidelines.xhtml

DISCUSSION

This study investigated characteristics of patients who died within three months of a diagnosis of colon cancer with the aim of identifying specific patient factors that can be addressed or accounted for to improve survival outcomes. Social isolation was identified as a common characteristic of early death in colon cancer patients. The different forms of social isolation studied included living alone and being unmarried (as opposed to co-habiting with a partner), residing in more deprived communities (as opposed to living in quintile 1-2 communities), living in a rural area when elderly (as opposed to an urban area) and having dementia or a psychiatric illness. Each of these factors was comparatively associated with early death.

Previous studies suggested that poorer outcomes for unmarried people and those living alone were mediated through weak social support[28], that exerted its influence on outcomes through later presentation[29]. This view is consistent with several other studies that reported more negative cancer beliefs[30], lower symptom awareness and greater perceived barriers to GP help-seeking among this group[31]. While weaker social support has previously been associated with later stage disease[32, 33], in this study, despite the collection of a range of detailed pathway and treatment variables, the association between marital status, accommodation status and early death remained unexplained. Other studies suggested biopsychosocial explanations for poorer cancer outcomes in unmarried cancer patients, including chronic stress[34] and weaker immune response[35]. Marital status and accommodation status have an important association with cancer and health outcomes generally[36], yet it is a research area that remains relatively under-investigated.

As in other studies, deprivation quintile was associated with early death, with a gradient in the odds of early death with increasing deprivation score[4, 37, 38]. However, unlike other studies this association seems restricted to women. Like marital / accommodation status, there was little evidence from this study suggesting that the association between deprivation

For peer review only - http://bmjopen.bmj.com/site/about/guidelines.xhtml

and early death is explained by characteristics of the pathway to diagnosis as the associations persisted after adjusting for attendance at A&E in the pathway to diagnosis, Dukes Stage and GP episodes in the three months to diagnosis. There is, of course, no direct biological basis for an association between deprivation and survival, mediating factors may include lower performance status due to higher tobacco consumption[39], lower uptake of treatments due to fatalistic cancer beliefs[40] or differential access to services[41]. The association between rural residence and early death among the oldest patients was independent of deprivation and strongly significant. This is likely to relate to either isolation, access to services or both. This is a well-defined target group for early diagnosis interventions but further study is required to investigate the link between lack of social contact and cancer survival if one exists.

Similar to previous work on early colorectal cancer death[4], a consistent feature associated with early death was that of incomplete data due to limited diagnostic testing. Missing histology, stage, grade and anatomical site may be explained by very ill patient's not receiving complete investigation. These characteristics may therefore be viewed as confounding by indication; as opposed to explaining early death, the associations are explained by early death.

The relationships between unknown marital, accommodation and outcomes are more difficult to explain. Despite the fact that GP records and secondary care databases were searched and the information relates to a period over a year prior to diagnosis of cancer, a strong association with early death was observed indicating the data was not missing at random. Missing data on living status, smoking status and alcohol consumption were also associated with early death in univariate analysis. Further work is required to explain this relationship with possible areas for investigation including the patient/practitioner relationship.

For peer review only - http://bmjopen.bmj.com/site/about/guidelines.xhtml

While co-morbidity was a common feature of patients in this study we did not find it associated with dying within three months of diagnosis. Previous studies have observed that co-morbidity exerted the greatest influence in the later phases of the survival pathway[42] though CCI score has been observed as an independent indicator of early death elsewhere[4]. We had matched cases and controls on age and as co-morbidity is strongly linked to age, this may have reduced our ability to detect this as an independent factor. In addition, being underweight had a strong independent association with early death. This was measured between one and three years before diagnosis and it is likely either related to disease progressing over a longer time period or to poorer performance status. However, as only 17 cases were described as underweight it explains less than 4% of the total early deaths.

While the CCI was not associated with early death, dementia and other psychiatric illness, as individual co-morbid conditions were associated with early death, though in multivariable analysis these relationships did not persist. Those with dementia were more likely to have missing data on stage and anatomical location, perhaps suggesting that these patients were less likely to be investigated for their disease. Similar findings have been reported in other colon cancer studies, with dementia associated with poorer colorectal cancer outcomes, later stage disease[43,] and less invasive investigation[44]. The relationship between other psychiatric illness and early death was also attenuated by stage again suggesting a role of diagnostic intervals on the pathway to diagnosis explaining early death. Underlying causes of delay may relate to symptom recognition by carer, patient, practitioner as well as patient communication or competing health care priorities.

A key objective of this study was to determine if patient health-seeking characteristics were associated with early death following colon cancer diagnosis. Uptake of the flu vaccine, baseline consultation activity and non-attendance at appointments were identified as three easily captured indicators of health-seeking behaviour. It was hypothesised that patients with

For peer review only - http://bmjopen.bmj.com/site/about/guidelines.xhtml

more regular or more compliant health-seeking behaviour would have better outcomes than those without, mediated through longer diagnostic intervals and later stage disease.

With regard to health seeking behaviour, although attendance for the flu vaccine was inversely associated with early death, this association was not significant in multivariable analysis. While attendance for the vaccine may be considered an indicator of health compliance, it is likely to reflect various health attitudes and behaviours, with previous UK studies reporting several social and cultural factors that were associated with flu vaccination uptake as well as perception regarding health status and susceptibility to flu[45]. The other indicator of health seeking, frequent GP attendance, was correlated with co-morbidity in this study; a relationship reported in several other research studies[10, 46].

Although ex-smokers were identified in univariate analysis as having non-significantly lower odds of early death, this association was largely explained in the minimally adjusted model with little evidence to suggest that poorer outcomes among smokers is real. While previous studies have demonstrated a significant association between alcohol-related hospital admissions and early death following a colorectal cancer diagnosis[38], this association was not observed in the current study possibly because this study was unable to discriminate between heavy and moderate alcohol use.

Strengths and weaknesses

The study sample was generated from a high-quality population-based cancer registry system with relatively few DCO cases[47] with full access to General Practice records and hospital clinical records. While missing data was a feature of the study, rather than acting as an impediment to our understanding of characteristics of early death, this appears to be one of the defining features of this patient group. Another recent study of early death has reported a similar pattern of high levels of missing data in those with the poorest outcomes[4].

For peer review only - http://bmjopen.bmj.com/site/about/guidelines.xhtml

However, the fact that remains that missing data was a feature of this study and potential improvements to the way data is recorded in the NICR are continuously being made.

The study investigated a broad range of factors that may be associated with early death and allowed for adjustment of a range of confounding factors such as co-morbidity, smoking and alcohol status.

The recording of the patient characteristics that vary over time at between one and three years before diagnosis was an important feature of the study design. Cancer diagnosis has been previously identified as being associated with changes in health-seeking behaviour, co-morbidity, BMI and lifestyle factors. Recording these factors based on over one year before diagnosis strengthens the assessment of causal inference between these variables and early death.

The case-control design in the study provided an efficient methodology to collect data and allowed the development of a control group that was matched on important but non-modifiable characteristics. While age, sex and year of diagnosis were fixed in the current study, their interaction with diagnostic pathway features and other characteristics could not be investigated. Previous studies have shown longer diagnostic timelines, later stage disease at diagnosis, lower symptom awareness and more negative cancer beliefs to be variable depending on age[48] and sex[49]. In addition, the matching on age may have reduced variation in other characteristics such as co-morbidity. The study population was also selected in a period before the introduction of screening, therefore all patients in both the case and control group were clinically diagnosed. While bowel screening was introduced in NI in 2011[23] and now represents an important pathway in cancer diagnosis, the majority of patients are still diagnosed clinically and the early clinical detection with symptoms remains both important and relevant.

For peer review only - http://bmjopen.bmj.com/site/about/guidelines.xhtml

The use of a control group of longer survivors provided useful comparative information to investigate risk of early death, with the choice of a control sample of deceased colon cancer patients removing any risk of consent bias from the study. However, while a buffer of three months was placed between cases and controls to allow better discrimination between the two, the survival of controls was restricted to a population of patients whose survival was less than three years and perhaps not that dissimilar to the case population. Despite this, the study was able to identify several characteristics which discriminated between cases and controls suggesting that the patients who die within the first few months of diagnosis are a specific patient cohort who require specific attention.

CONCLUSIONS

This comprehensive study of early death from colon cancer has identified several population sub-groups that warrant special attention. These include those who are single, living alone, older people living in rural environments, and people from the most deprived communities as well as those living in residential or nursing care. These likely comprise some of the most isolated people in society. However, while the aforementioned variables are an indicator of social isolation, this study was not designed to actually *investigate* isolation (i.e. lack of social contact or poor social support networks). Therefore, further study is required to confirm that social isolation is definitely linked with poor cancer survival outcomes. In addition, further studies are required to better understand the role of missing data in patient records. Finally, additional work ought to be undertaken to determine if these patterns are consistent in other ICBP countries.

For peer review only - http://bmjopen.bmj.com/site/about/guidelines.xhtml

ACKNOWLEDGEMENTS

The authors gratefully appreciate the contribution of data abstractors: Donna Floyd, Rosemary Ward, Jacqui Napier, Kate Donnelly and Bríd Morris. The authors also thank Business Services Organisation, Health & Social Care NI for facilitating the note review.

FUNDING

The TEDI study was funded by the Cancer Research UK led National Awareness & Early Diagnosis Initiative. The Northern Ireland Cancer Registry is funded by the Public Health Agency Northern Ireland.

DATA SHARING

Data from the TEDI study is available from the N. Ireland Cancer Registry with approval from the data custodian. Interested parties should contact Dr Anna Gavin, Director, N. Ireland Cancer Registry.

COMPETING INTERESTS

There are no competing interests to be reported by the authors in this study

CONTRIBUTOR STATEMENT

CD designed the study, undertook all statistical analysis and drafted the manuscript. NH was medical adviser for the study, provided advice on statistical analysis and interpretation and contributed to the writing of the manuscript. AM contributed to the writing, editing and submission of the paper. MD provided advice and guidance on the study design and contributed to writing of the manuscript. LA provided advice and guidance on the study design and contributed to writing of the manuscript. LR contributed to the study design, development of data collection forms and data abstractor training and analysis and

For peer review only

interpretation of results. AG was Principal Investigator, funding recipient and contributed to the writing of the manuscript.

REFERENCES

1 Coleman MP, Forman D, Bryant H, Butler J, Rachet B, Maringe C, Nur U, Tracey E, Coory M, Hatcher J, McGahan CE, Turner D, Marrett L, Gjerstorff ML, Johannesen TB, Adolfsson J, Lambe M, Lawrence G, Meechan D, Morris EJ, Middleton R, Steward J, Richards MA; ICBP Module 1 Working Group. Cancer survival in Australia, Canada, Denmark, Norway, Sweden, and the UK, 1995-2007 (the International Cancer Benchmarking Partnership): an analysis of population-based cancer registry data. Lancet. 2011 Jan 8;377(9760):127-38.

2 De Angelis, R., Sant, M., Coleman, M.P., Francisci, S., Baili, P., Pierannunzio, D., Trama, A., Visser, O., Brenner, H., Ardanaz, E. and Bielska-Lasota, M., 2014. Cancer survival in Europe 1999–2007 by country and age: results of EUROCARE-5—a population-based study. *The lancet oncology*, *15*(1), pp.23-34.

3 Morris EJ, Sandin F, Lambert PC, Bray F, Klint A, Linklater K, Robinson D, Påhlman L, Holmberg L, Møller H. A population-based comparison of the survival of patients with colorectal cancer in England, Norway and Sweden between 1996 and 2004. Gut. 2011 Feb 8.

4 Downing, A., Aravani, A., Macleod, U., Oliver, S., Finan, P.J., Thomas, J.D., Quirke, P., Wilkinson, J.R. and Morris, E.J.A., 2013. Early mortality from colorectal cancer in England: a retrospective observational study of the factors associated with death in the first year after diagnosis. British journal of cancer, 108(3), pp.681-685.

5 Northern Ireland Cancer Registry. Official Statistics on cancer incidence and survival: All Cancers (Excluding Non Melanoma Skin Cancer) 2015. http://www.qub.ac.uk/researchcentres/nicr/FileStore/PDF/FactSheets/Filetoupload,531393,en.pdf.

For peer review only - http://bmjopen.bmj.com/site/about/guidelines.xhtml

6 Thomson C S, Forman D. Cancer survival in England and the influence of early diagnosis: what can we learn from recent EUROCARE results?. Br J Cancer. 2009 December 3; 101(Suppl 2): S102–S109.

7 Sogaard, M., Thomsen, R.W., Bossen, K.S., Sorensen, H.T. and Norgaard, M., 2013. The impact of comorbidity on cancer survival: a review. Clin Epidemiol, 5(Suppl 1), pp.3-29.

8 Genevieve M. Boland, George J. Chang, Alex B. Haynes, Yi-Ju Chiang, Ryaz Chagpar, Yan Xing, Chung-Yuan Hu, Barry W. Feig, Y. Nancy You, Janice N. Cormier. Association between Adherence to National Comprehensive Cancer Network Treatment Guidelines and Improved Survival in Patients with Colon Cancer. Cancer. 2013 April 15; 119(8): 1593–1601.

9 Mitchell, E., Macdonald, S., Campbell, N.C., Weller, D. and Macleod, U., 2008. Influences on pre-hospital delay in the diagnosis of colorectal cancer: a systematic review. British journal of cancer, 98(1), pp.60-70.

10 Marshall JR, Funch DP (1986) Gender and illness behavior among colorectal cancer patients. Women and Health 11(3 – 4): 67 – 82

11 Mariscal M, Llorca J, Prieto D, Delgado-Rodriguez M (2001) Determinants of the interval between the onset of symptoms and diagnosis in patients with digestive tract cancers. Cancer Detect Prevent 25(5): 420 – 429

12 McCaffery K, Wardle J, Waller J (2003) Knowledge, attitudes, and behavioural intentions in relation to the early detection of colorectal cancer in the United Kingdom. Prev Med 36: 525 – 535

13 Robinson E, Mohilever J, Zidan J, Sapir D (1986) Colorectal cancer: incidence, delay in diagnosis and stage of disease. Eur J Cancer Clin Oncol 22(2): 157 – 161

14 Bain NSC, Campbell NC, Ritchie LD, Cassidy J (2002) Striking the right balance in colorectal cancer – a qualitative study of rural and urban patients. Fam Pract 19(4): 369 – 374

For peer review only - http://bmjopen.bmj.com/site/about/guidelines.xhtml

15 Holliday HW, Hardcastle JD (1979) Delay in diagnosis and treatment of symptomatic colorectal cancer. Lancet 1: 309 – 311

16 MacAdam DB (1979) A study in general practice of the symptoms and delay patterns in the diagnosis of gastrointestinal cancer. J R Coll Gen Pract 29: 723 – 729

17 Mor V, Masterson-Allen S, Goldberg R, Guadagnoli E, Wool MS (1990) Prediagnostic symptom recognition and help-seeking among cancer patients. J Community Health 15(4): 253 – 266

18 Curless R, French JM, Williams GV, James OFW (1994) Colorectal carcinoma: do elderly patients present differently? Age Ageing 23: 102 – 107

19 Mitchell E, Macdonald S, Campbell NC, Weller D, Macleod U (2008) Influences on pre-hospital delay in the diagnosis of colorectal cancer: a systematic review. Br J Cancer 98: 60 – 70

20 Nilsson E, Bolin S, Sjodahl R (1982) Carcinoma of the colon and rectum. Delay in diagnosis. Acta Chir Scand 148: 617 – 622

21 Roncoroni L, Pietra N, Violi V, Sarli L, Choua O, Peracchia A (1999) Delay in the diagnosis and outcome of colorectal cancer: a prospective study. Eur J Surg Oncol 25: 173 – 178

22 Weller, D., Coleman, D., Robertson, R., Butler, P., Melia, J., Campbell, C., Parker, R., Patnick, J. and Moss, S., 2007. The UK colorectal cancer screening pilot: results of the second round of screening in England. *British journal of cancer*, *97*(12), pp.1601-1605.

23 Bradley DT, Treanor C, McMullan C, Owen T, Graham A, Anderson D. Reasons for non-participation in the Northern Ireland Bowel Cancer Screening Programme: a qualitative study. BMJ Open. 2015 Sep 9;5(9).

24 Elliss-Brookes, L., McPhail, S., Ives, A., Greenslade, M., Shelton, J., Hiom, S. and Richards, M., 2012. Routes to diagnosis for cancer–determining the patient journey using multiple routine data sets. British Journal of Cancer, 107(8), pp.1220-1226.

25 Weller, D., Vedsted, P., Rubin, G., Walter, F.M., Emery, J., Scott, S., Campbell, C., Andersen, R.S., Hamilton, W., Olesen, F. and Rose, P., 2012. The Aarhus statement:

For peer review only - http://bmjopen.bmj.com/site/about/guidelines.xhtml

improving design and reporting of studies on early cancer diagnosis. British Journal of Cancer, 106(7), pp.1262-1267.

26 Lunt M. Optimal matching for case control studies, Computer program and documentation for use with Stata statistical software. Available at http://personalpages.manchester.ac.uk/staff/mark.lunt/optmatch2.hlp; 2007. p. 1.2, Accessed 24/12/2011.

27 Rosenbaum P.R., Optimal matching for observational studies, J Am Stat Assoc (1989), pp. 1024–1032.

28 Villingshøj M, Ross L, Thomsen BL, Johansen C. Does marital status and altered contact with the social network predict colorectal cancer survival? Eur J Cancer. 2006 Nov;42(17):3022-7.

29 Mitchell, E.D., Pickwell-Smith, B. and Macleod, U., 2015. Risk factors for emergency presentation with lung and colorectal cancers: a systematic review. BMJ open, 5(4), p.e006965.

30 Quaife, S.L., Winstanley, K., Robb, K.A., Simon, A.E., Ramirez, A.J., Forbes, L.J., Brain, K.E., Gavin, A. and Wardle, J., 2015. Socioeconomic inequalities in attitudes towards cancer: an international cancer benchmarking partnership study. European Journal of Cancer Prevention, 24(3), p.253.

31 Niksic, M., Rachet, B., Warburton, F.G., Wardle, J., Ramirez, A.J. and Forbes, L.J.L., 2015. Cancer symptom awareness and barriers to symptomatic presentation in England—are we clear on cancer?. British journal of cancer, 113(3), pp.533-542.

32 Aizer AA, Chen MH, McCarthy EP, Mendu ML, Koo S, Wilhite TJ, Graham PL, Choueiri TK, Hoffman KE, Martin NE, Hu JC, Nguyen PL. Marital status and survival in patients with cancer. J Clin Oncol. 2013;31:3869–3876.

33 Nayeri K, Pitaro G, Feldman JG. Marital status and stage at diagnosis in cancer. New York state journal of medicine. 1992;92:8–11.

For peer review only - http://bmjopen.bmj.com/site/about/guidelines.xhtml

34 Levy SM, Herberman RB, Whiteside T, Sanzo K, Lee J, Kirkwood J. Perceived social support and tumor estrogen/progesterone receptor status as predictors of natural killer cell activity in breast cancer patients. Psychosomatic medicine. 1990;52:73–85.

35 Hamilton TD, Leugner D, Kopciuk K, Dixon E, Sutherland FR, Bathe OF. Identification of prognostic inflammatory factors in colorectal liver metastases. BMC Cancer. 2014;14:542.

36 Berkman, L.F., 1995. The role of social relations in health promotion. Psychosomatic medicine, 57(3), pp.245-254

37 Henrik Møller, Fredrik Sandin, David Robinson, Freddie Bray, d, Åsa Klint, Karen M. Linklater, Paul C. Lambert, g, Lars Påhlmanh, Lars Holmberg, j, Eva Morris. Colorectal cancer survival in socioeconomic groups in England: Variation is mainly in the short term after diagnosis. Eur J Cancer. Volume 48, Issue 1, January 2012, Pages 46–53.

38 D H Brewster, D I Clark, D L Stockton, A J Munro, R J C Steele. Characteristics of patients dying within 30 days of diagnosis of breast or colorectal cancer in Scotland, 2003–2007. Br J Cancer. 2011 January 4; 104(1): 60–67.

39 Munro, A.J., Bentley, A.H.M., Ackland, C. and Boyle, P.J., 2006. Smoking compromises cause-specific survival in patients with operable colorectal cancer. Clinical Oncology, 18(6), pp.436-440.

40 Beeken, R.J., Simon, A.E., von Wagner, C., Whitaker, K.L. and Wardle, J., 2011. Cancer fatalism: deterring early presentation and increasing social inequalities?. *Cancer Epidemiology Biomarkers & Prevention*, *20*(10), pp.2127-2131.

41 Raine, R., Wong, W., Scholes, S., Ashton, C., Obichere, A. and Ambler, G., 2010. Social variations in access to hospital care for patients with colorectal, breast, and lung cancer between 1999 and 2006: retrospective analysis of hospital episode statistics. *Bmj*, *340*, p.b5479.

For peer review only - http://bmjopen.bmj.com/site/about/guidelines.xhtml

42 Sogaard, M., Thomsen, R.W., Bossen, K.S., Sorensen, H.T. and Norgaard, M., 2013. The impact of comorbidity on cancer survival: a review. Clin Epidemiol, 5(Suppl 1), pp.3-29.

43 Gupta SK, Lamont EB. Patterns of presentation, diagnosis, and treatment in older patients with colon cancer and comorbid dementia. J Am Geriatr Soc 2004; 52:1681–1687.

44 Raji, M.A., Kuo, Y.F., Freeman, J.L. and Goodwin, J.S., 2008. Effect of a dementia diagnosis on survival of older patients after a diagnosis of breast, colon, or prostate cancer: implications for cancer care. Archives of internal medicine, 168(18), pp.2033-2040.

45 Nagata, J.M., Hernández-Ramos, I., Kurup, A.S., Albrecht, D., Vivas-Torrealba, C. and Franco-Paredes, C., 2013. Social determinants of health and seasonal influenza vaccination in adults≥ 65 years: a systematic review of qualitative and quantitative data. BMC Public Health, 13(1), p.1.

46 van Oostrom, S.H., Picavet, H.S.J., de Bruin, S.R., Stirbu, I., Korevaar, J.C., Schellevis, F.G. and Baan, C.A., 2014. Multimorbidity of chronic diseases and health care utilization in general practice. BMC family practice, 15(1), p.1.

47 Kearney, TM, Donnelly, C, Kelly, JM, O'Callaghan, EP, Fox, CR & Gavin, AT 2015, 'Validation of the completeness and accuracy of the Northern Ireland Cancer Registry' Cancer Epidemiology, vol 39, no. 3, pp. 401-404.

48 Forbes LJ, Simon AE, Warburton F, Boniface D, Brain KE, Dessaix A, Donnelly C, Haynes K, Hvidberg L, Lagerlund M, Lockwood G, Tishelman C, Vedsted P, Vigmostad MN, Ramirez AJ, Wardle J; International Cancer Benchmarking Partnership Module 2 Working Group. Differences in cancer awareness and beliefs between Australia, Canada, Denmark, Norway, Sweden and the UK(the International Cancer Benchmarking Partnership): do they contribute to differences in cancer survival? Br J Cancer. 2013 Feb 5;108(2):292-300.

For peer review only - http://bmjopen.bmj.com/site/about/guidelines.xhtml

For peer review only

49 Evans REC, Brotherstone H, Miles A, Wardle J. Gender differences in early detection of cancer. J Mens Health Gender. 2005;2 (2:209–217).

FIGURE LEGEND

Figure 1: case inclusions and exclusions

For peer review only

For peer review only - http://bmjopen.bmj.com/site/about/guidelines.xhtml

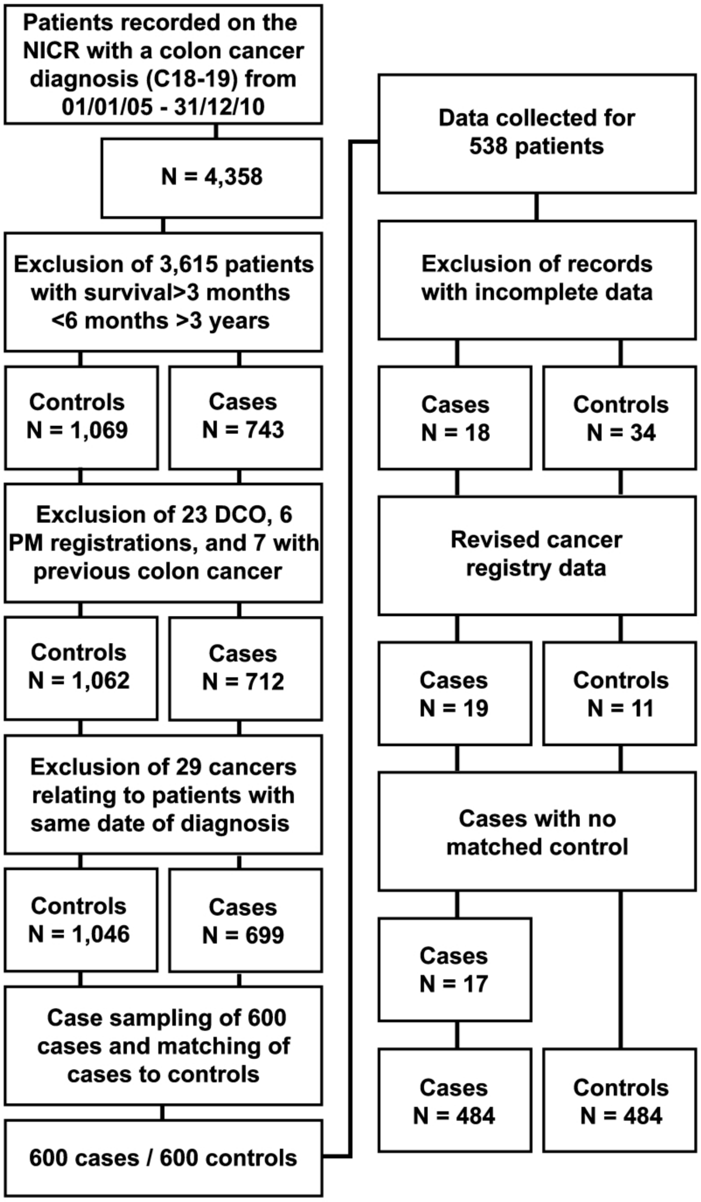

Case inclusions and exclusions

144x247mm (600 x 600 DPI)

Supplementary Table S1: Distribution of the case sample and the remaining patients who died within three months of diagnosis who were not included in the study

Characteristics		Case Sample n=484	Cases not included n=196	P value
Sex %	Male	52.7	44.3	
	Female	47.3	55.7	0.05$^{\chi}$
Year diagnosed %	2005-06	39.2	29.4	
	2007-08	38.5	18.40	
	2009-10	22.3	51.3	<0.01$^{\chi}$
Dukes stage %	A	2.3	1.0	
	B	10.1	7.0	
	C	10.7	9.0	
	D	37.0	44.3	
	Unknown	39.9	38.8	0.27$^{\chi}$
Deprivation quintile %	1 (least deprived)	16.6	15.2	
	2	20.9	22.7	
	3	17.4	18.7	
	4	24.8	21.7	
	5 (most deprived)	20.3	21.7	0.87$^{\chi}$
Mean age at diagnosis in years (Standard deviation)		76.5 (10.6)	77.6 (10.3)	0.83†
Mean survival duration in days (Standard deviation)		36.7 (24.7)	33.8 (39.9)	0.82$^{\beta}$

P-values presented for chi squared tests (χ), t-tests (†) and Kaplan Meier (β)

For peer review only

STROBE 2007 (v4) checklist of items to be included in reports of observational studies in epidemiology*
Checklist for cohort, case-control, and cross-sectional studies (combined)

Section/Topic	Item #	Recommendation	Reported on page #
**Title and abstract**	1	(*a*) Indicate the study's design with a commonly used term in the title or the abstract	1
		(*b*) Provide in the abstract an informative and balanced summary of what was done and what was found	2
**Introduction**			
Background/rationale	2	Explain the scientific background and rationale for the investigation being reported	4
Objectives	3	State specific objectives, including any pre-specified hypotheses	5
**Methods**			
Study design	4	Present key elements of study design early in the paper	2 & 5
Setting	5	Describe the setting, locations, and relevant dates, including periods of recruitment, exposure, follow-up, and data collection	2 & 5
Participants	6	(*a*) *Cohort study*—Give the eligibility criteria, and the sources and methods of selection of participants. Describe methods of follow-up<br>*Case-control study*—Give the eligibility criteria, and the sources and methods of case ascertainment and control selection. Give the rationale for the choice of cases and controls<br>*Cross-sectional study*—Give the eligibility criteria, and the sources and methods of selection of participants	5
		(*b*) *Cohort study*—For matched studies, give matching criteria and number of exposed and unexposed<br>*Case-control study*—For matched studies, give matching criteria and the number of controls per case	5
Variables	7	Clearly define all outcomes, exposures, predictors, potential confounders, and effect modifiers. Give diagnostic criteria, if applicable	6
Data sources/ measurement	8*	For each variable of interest, give sources of data and details of methods of assessment (measurement). Describe comparability of assessment methods if there is more than one group	*6*
Bias	9	Describe any efforts to address potential sources of bias	6-7
Study size	10	Explain how the study size was arrived at	6
Quantitative variables	11	Explain how quantitative variables were handled in the analyses. If applicable, describe which groupings were chosen and why	6-7
Statistical methods	12	(*a*) Describe all statistical methods, including those used to control for confounding	6-7
		(*b*) Describe any methods used to examine subgroups and interactions	7
		(*c*) Explain how missing data were addressed	6, 16
		(*d*) *Cohort study*—If applicable, explain how loss to follow-up was addressed<br>*Case-control study*—If applicable, explain how matching of cases and controls was addressed	5

For peer review only

		Cross-sectional study—If applicable, describe analytical methods taking account of sampling strategy	
		(*e*) Describe any sensitivity analyses	-
**Results**			
Participants	13*	(a) Report numbers of individuals at each stage of study—eg numbers potentially eligible, examined for eligibility, confirmed eligible, included in the study, completing follow-up, and analysed	4
		(b) Give reasons for non-participation at each stage	Fig 1
		(c) Consider use of a flow diagram	Fig 1
Descriptive data	14*	(a) Give characteristics of study participants (eg demographic, clinical, social) and information on exposures and potential confounders	Table 1
		(b) Indicate number of participants with missing data for each variable of interest	Table 1-4
		(c) *Cohort study*—Summarise follow-up time (eg, average and total amount)	
Outcome data	15*	*Cohort study*—Report numbers of outcome events or summary measures over time	
		Case-control study—Report numbers in each exposure category, or summary measures of exposure	Table 1-4
		Cross-sectional study—Report numbers of outcome events or summary measures	
Main results	16	(*a*) Give unadjusted estimates and, if applicable, confounder-adjusted estimates and their precision (eg, 95% confidence interval). Make clear which confounders were adjusted for and why they were included	Table 1-6
		(*b*) Report category boundaries when continuous variables were categorized	Table 1-4
		(*c*) If relevant, consider translating estimates of relative risk into absolute risk for a meaningful time period	
Other analyses	17	Report other analyses done—eg analyses of subgroups and interactions, and sensitivity analyses	Table 5
**Discussion**			
Key results	18	Summarise key results with reference to study objectives	15
Limitations	19	Discuss limitations of the study, taking into account sources of potential bias or imprecision. Discuss both direction and magnitude of any potential bias	18-19
Interpretation	20	Give a cautious overall interpretation of results considering objectives, limitations, multiplicity of analyses, results from similar studies, and other relevant evidence	19-20
Generalisability	21	Discuss the generalisability (external validity) of the study results	19
**Other information**			
Funding	22	Give the source of funding and the role of the funders for the present study and, if applicable, for the original study on which the present article is based	21

*Give information separately for cases and controls in case-control studies and, if applicable, for exposed and unexposed groups in cohort and cross-sectional studies.

Note: An Explanation and Elaboration article discusses each checklist item and gives methodological background and published examples of transparent reporting. The STROBE checklist is best used in conjunction with this article (freely available on the Web sites of PLoS Medicine at http://www.plosmedicine.org/, Annals of Internal Medicine at http://www.annals.org/, and Epidemiology at http://www.epidem.com/). Information on the STROBE Initiative is available at www.strobe-statement.org.

For peer review only

Predictors of an early death in patients diagnosed with colon cancer: a retrospective case-control study in the United Kingdom

Journal:	*BMJ Open*
Manuscript ID	bmjopen-2018-026057.R1
Article Type:	Research
Date Submitted by the Author:	20-Mar-2019
Complete List of Authors:	Donnelly, Conan; National Cancer Registry Ireland, University of Cork<br>Hart, Nigel; Queen's University Belfast, School of Medicine, Dentistry and Biomedical Sciences - Centre for Public Health<br>McCrorie, Alan; Queen's University Belfast, Centre for Cancer Research and Cell Biology<br>Donnelly, Michael; Queen's University Belfast, Centre for Public Health<br>Anderson, Lesley; Queen's University Belfast, School of Medicine<br>Ranaghan, Lisa; Belfast Health and Social Care Trust<br>Gavin, Anna; Queen's University Belfast, N. Ireland Cancer Registry
**Primary Subject Heading**:	Epidemiology
Secondary Subject Heading:	Public health
Keywords:	Epidemiology < ONCOLOGY, STATISTICS & RESEARCH METHODS, Gastrointestinal tumours < ONCOLOGY, Colorectal surgery < SURGERY

SCHOLARONE™ Manuscripts

Conan Donnelly[1*], Nigel Hart[2], Alan D McCrorie[3] Michael Donnelly[4], Lesley A Anderson[4], Lisa Ranaghan[5], Anna Gavin[1]

1. N. Ireland Cancer Registry, Queen's University Belfast, 2. Department of General Practice, Queen's University Belfast, 3. School of Medicine, Dentistry and Biomedical Science, Queen's University Belfast. 4. Centre for Public Health, Queen's University Belfast. 5. Belfast, Health and Social Care Trust.

*Corresponding author: c.donnelly@nicr.ie, N.Ireland Cancer Registry, Centre for Public Health, Queen's University Belfast, Grosvenor Road, Belfast, BT12 6DP

TITLE: Predictors of an early death in patients diagnosed with colon cancer: a retrospective case-control study in the United Kingdom

(Running title: Predictors of an early death from colon cancer)

KEY WORDS

Colon Cancer, Survival, health seeking, early death, early diagnosis

FUNDING

The TEDI study was funded by the Cancer Research UK led National Awareness & Early Diagnosis Initiative (Principal Investigator: AG, Co-Investigators: CD, NH, AM, MD, LR LA). The Northern Ireland Cancer Registry is funded by the Public Health Agency Northern Ireland (Principal Investigator: AG)

COMPETING INTERESTS

There are no competing interests to be reported by the authors in this study

WORD COUNT: 3865

FIGURES: 1, TABLES: 5, SUPPLEMENTARY TABLES: 1

For peer review only - http://bmjopen.bmj.com/site/about/guidelines.xhtml

ABSTRACT

OBJECTIVE: Despite considerable improvements, five-year survival rates for colon cancer in the United Kingdom (UK) remain poor when compared with other socioeconomically similar countries. Variation in five-year survival can be partly explained by higher rates of death within three months of diagnosis in the UK. This study investigated characteristics of patients who died within three months of a diagnosis of colon cancer with the aim of identifying specific patient factors that can be addressed or accounted for to improve survival outcomes.

DESIGN: A retrospective case-control study design was applied with matching on age, sex and year diagnosed. Patient, disease, clinical, and service characteristics of patients diagnosed with colon cancer in a UK region (2005-2010), who survived less than three months from diagnosis (cases) were compared with patients who survived between six and thirty-six months (controls). Patient and clinical data was sourced from General Practice notes and hospital databases 1-3 years pre-diagnosis.

RESULTS: Being older (aged ≥78 years) and living in deprivation quintile 5 (OR=2.64, CI: 1.15-6.06), being unmarried and living alone (OR=1.64, CI: 1.07-2.50), being underweight compared to normal weight or obese (OR=3.99, CI: 1.14-14.0) and being older and living in a rural as opposed to urban area (OR=1.96, CI: 1.21-3.17) were all independent predictors of early death from colon cancer. Missing information was also associated with early death including unknown stage, histological type and marital/accommodation status after accounting for other factors.

CONCLUSION: Several factors typically associated with social isolation were a recurring theme in patients who died early from colon cancer death. This association is unexplained by clinical or diagnostic pathway characteristics. Socially isolated patients are a key target group to improve outcomes of the worst surviving patients, but further investigation is required to determine if being isolated itself is actually cause of early death from colon cancer.

For peer review only - http://bmjopen.bmj.com/site/about/guidelines.xhtml

Strengths and limitations of this study

- The study sample was generated from a high-quality population-based cancer registry system with relatively few death certificate only (DCO) cases
- Case-control design provided an efficient method of collecting data and allowed the development of a control group that was matched on important non-modifiable characteristics
- Data used in this study predates the introduction of the national bowel cancer screening programme in this UK region which should mitigate any improvement in survival independently associated with bowel cancer screening
- Survival of controls was restricted to a population of patients whose survival was less than three years and similar to the case population
- Study identified several characteristics which discriminated between cases and controls suggesting that patients who die within the first few months of diagnosis are a specific patient cohort who require attention

For peer review only

For peer review only - http://bmjopen.bmj.com/site/about/guidelines.xhtml

INTRODUCTION

Despite considerable improvements, United Kingdom (UK) survival rates for colon cancer remain poor by international comparison with higher five-year survival reported in Norway, Sweden, Canada and Australia[1] and poorer survival in the UK compared to several countries reported in Eurocare[2]. These deficits have largely been explained by survival at three months post diagnosis[3]. Patients who survive beyond this period in the UK have similar five-year survival rates to their counterparts in better performing countries[1]. Approximately 19% of colorectal cancer patients in the UK and 16% in the Northern Ireland (NI) died within three months of diagnosis between 2006 and 2008[4-5]. It was estimated that if survival in England matched that of Norway, 13.6% fewer patients would die within the three-month period[3]. Generally poor survival is linked with a number of factors including late stage disease at diagnosis[6], poor patient fitness due to coexisting disease[7] and limited availability of and access to high quality investigations and treatment[8].

Reasons for diagnostic delay in colorectal cancer are well documented[9]. Lower educational status[10-11] and rural residence[12-13] have been associated with delayed help seeking. Additionally, stronger social networks have been associated with shorter diagnostic delay[14-16]. Clinical characteristics also play a role. Patients with co-morbid disease[11,17] and/or multiple symptoms[11] are less likely to delay compared to those with non-specific symptoms[17-18]. Application of referral guidelines by General Practitioners (GPs) has been shown to reduce delay[19] while younger patients[13,20], those of lower socio-economic status[21] and frequent help seekers[10,16] were less likely to be referred. While bowel cancer screening was introduced in the UK in 2007[22] and in NI in 2011[23], the vast majority patients are diagnosed clinically[24], therefore the role of clinical decision making in early colon cancer diagnosis remains paramount.

The relationship between these factors and surviving past the first few months following a colon cancer diagnosis has not been adequately investigated and their role in explaining international

For peer review only - http://bmjopen.bmj.com/site/about/guidelines.xhtml

survival differences requires attention. The aim of this study was to investigate patient, clinical and disease factors associated with early death in colon cancer patients in Northern Ireland and to determine factors which might help to identify subgroups in the population for early diagnosis interventions.

METHODOLOGY

This study employed a retrospective, individually matched, case-control design involving a posthumous review of primary care physician or GP and electronic secondary care notes. The study design was guided by the principles of the Aarhus statement on early diagnosis research[25]. Principles adhered to in this study include items 1-4, 7-9, and 20 of the Aarhus checklist. Date of initial cancer diagnosis is defined by the Northern Ireland Cancer Registry (NICR) as date of first tissue diagnosis in secondary care, not as symptom presentation in primary and/or secondary care.

Case and control definition and identification

Cases: patients diagnosed with primary colon cancer (ICD 10: C18) in Northern Ireland between January 2005 and December 2010 (prior to introduction of national bowel cancer screening programme in this region) were identified using the NICR. Using death registrations, provided by the General Registrar Office, the status and survival of patients was determined. Cases were defined as patients with an observed survival of under 90 days following diagnosis date (as assigned by NICR). A random sample of all eligible cases was selected using random number tables based on pre-defined power calculations.

Controls: patients with an observed survival lasting over six months and less than three years leaving a three-month buffer between the survival rates of cases and controls. Controls were individually matched, using individual nearest neighbour matching[26-27] to cases by age (within 5-year age bands), sex and year of diagnosis (within 2-year groups). In both groups, patients

For peer review only - http://bmjopen.bmj.com/site/about/guidelines.xhtml

with incident cancer identified by death certificate only (DCO) and patients with recurrence of a previous incident colon cancer were excluded.

Exposure variables and covariates

Data items were identified through literature review with items and categorisation defined in consultation with a clinical adviser, GP, a colorectal surgeon and an oncologist. Items were classified into seven areas: demographic factors acting as a surrogate for social isolation (marital status, accommodation status, NI Multiple Deprivation Measure [NI MDM quintile], rural/urban status), lifestyle (smoking and alcohol status, health seeking activity including uptake of flu vaccine and frequency of GP attendance) and co-morbidities (Charlson Co-Morbidity score [CCI] and psychiatric illnesses). These characteristics were collected from information recorded between one and three years before diagnosis. Marital and accommodation status were merged in final analysis due to multicollinearity.

Disease characteristics included symptoms in the year to diagnosis, disease stage at diagnosis with histology, morphology and grade collected from pathology records held in the NICR. GP and hospital episodes [including symptoms (classified as 'vague' or 'alarm' based on NICE Guidelines for Suspected Cancer Referral guidelines), clinician actions (number of GP episodes before diagnosis and referral) and investigations ordered]. In addition, treatment [first treatment type, treatment intent, surgical resection (y/n), radiotherapy (y/n), chemotherapy (y/n)] and death information (date, place and cause of death) were also collected. Data were collected by two trained data abstractors under the guidance of a medically trained clinical adviser using a common bespoke proforma. Data was sourced from GP records, electronic hospital records including the hospital discharge records, multidisciplinary team (MDT) and oncology data systems. Assuming β=0.8 and a 2-sided test with a significance level of 5%, a sample size of N=960 (480 cases matched to 480 controls) sufficiently powers this study to detect an odds ratio of 2.1 for any risk factor with a prevalence

For peer review only - http://bmjopen.bmj.com/site/about/guidelines.xhtml

of 5%, an odds ratio of 1.8 for any risk factor with a prevalence of 10%, and an odds ratio of 1.6 for any risk factor with a prevalence of 15%.

Statistical method

Data was analysed using 'STATA 14 (StataCorp 2015)'. All missing data were categorised as unknown and included in the analysis. Univariate analysis involved cross-tabulation of all categorical variables with case/control status. Conditional logistic regression (CLR) was used to produce unadjusted odds ratios and associated 95% confidence intervals to identify independent factors associated with early death. Patient characteristics that were deemed to be clinically significant and/or statistically significant at the p<0.25, were included in a minimally adjusted multivariable model to test independence from other co-morbidities, patient and disease characteristics. Stage and pathway to diagnosis characteristics (number of A&E and GP episodes in three months preceding diagnosis) were added to the models to assess the degree to which they explained variation in early death among different patient groups. Age (a binary classification around median age [78 years; IQR = 19] of cases) and sex stratified univariate and multivariable analysis were undertaken to investigate differences in patterns in early death between these groups.

Patient and public involvement

Members of the public including patients were not involved in the design or analysis stages of this piece of non-interventional research, but research question was designed to explore characteristics of patients who die early after a diagnosis of colon cancer. Ethical approval for this study was granted by the Office of Research Ethics Committees Northern Ireland (12/NI/0034). This committee receives input from lay member(s) of the public before reaching a decision about whether or not to approve research studies.

For peer review only - http://bmjopen.bmj.com/site/about/guidelines.xhtml

RESULTS

There were 4,358 colon cancer tumours between 2005 and 2010 registered by the NICR. Of these, 743 (17%) related to patients who died within 3 months of diagnosis and 1,069 related to patients who died between six months and three years. Following exclusions and sampling (Figure 1), 484 cases and the same number of matched controls were generated. There were no significant differences between cases included in the study and those not included (Supplementary Table 1) regarding stage at diagnosis, deprivation quintile, age and survival. However, the study group included significantly more males than females as well as fewer patients diagnosed in 2009 and 2010 due to resource constraints in data collection.

Univariate analysis

Compared to married patients, odds of early death were higher among single, widowed and those with unknown marital status (Table 1). Those who lived alone, in nursing or residential care or were living with another relative were more likely to die within three months compared to those living with a spouse/partner. Odds of early death were also higher in the most deprived communities (23%) compared to the least deprived (13%) (Table 1).

Table 1: Demographic characteristics of cases and controls and associated odds ratios for early death and 95% confidence intervals

Characteristic		Case		Control		OR†	95% CI
		n	%	n	%		
Accommodation status	Spouse/ partner	156	32.2	233	48.1	1	
	Nursing/residential	48	9.9	23	4.8	3.93	2.18 – 7.09
	Sheltered dwelling	8	1.7	14	2.9	0.91	0.37 – 2.24
	Alone	156	32.2	152	31.4	1.74	1.23 – 2.45
	Lives with relative	53	11.0	40	8.3	2.32	1.41 – 3.82
	Unknown	63	13.0	22	4.6	5.32	3.00 – 9.43
Marital status	Married/Cohabiting	189	39.1	257	53.1	1	

For peer review only - http://bmjopen.bmj.com/site/about/guidelines.xhtml

	Divorced	15	3.1	13	2.7	1.51	0.66 – 3.45
	Unknown	44	9.1	15	3.1	3.79	2.06 – 6.96
	Single	74	15.3	53	11.0	1.88	1.25 – 2.84
	Widowed	162	33.5	146	30.2	1.60	1.14 – 2.22
Urban/rural status	Rural	178	63.2	166	65.7	1	
	Urban	306	36.8	318	34.3	0.89	0.68 – 1.17
Deprivation quintile	Q1 (least deprived)	64	13.2	89	18.4	1	
	Q2	95	19.6	92	19.0	1.45	0.76 – 1.74
	Q3	106	21.9	111	22.9	1.01	0.66 – 1.54
	Q4	110	22.7	102	21.1	1.49	0.99 – 2.24
	Q5 (most deprived)	109	22.5	90	18.6	1.47	0.95 - 2.27
Influenza vaccination uptake	No uptake	71	14.7	47	9.7	1	
	≥1 vaccination	324	66.9	345	71.3	0.63	0.43-0.94
	Unknown	89	18.4	92	19.0	0.65	0.40-1.05
Baseline consultation activity (tercile)	<11	149	30.8	156	32.2	1	
	11-19	148	30.6	162	33.5	0.98	0.71-1.34
	≥20	187	38.6	166	34.3	1.20	0.87-1.67
Smoking	Non-smoker	221	45.7	219	15.7	1	
	Ex-smoker	141	29.1	175	36.2	0.76	0.55 – 1.04
	Current smoker	91	18.8	76	15.7	1.19	0.81 – 1.75
	Unknown	31	6.4	14	2.9	2.30	1.16 – 4.56
Alcohol Consumption	Current drinker	154	31.8	175	36.2	1	
	Ex-drinker	31	6.4	26	5.4	1.37	0.78 – 2.43
	Never drank	189	39.1	191	39.5	1.13	0.82 – 1.55
	Unknown	110	22.7	92	19.0	1.38	0.96 – 1.99

† Conditional logistic regression odds ratios for cases and controls matched on age, sex and year diagnosed

For peer review only - http://bmjopen.bmj.com/site/about/guidelines.xhtml

Baseline GP consultation activity was not associated with early death. Regarding influenza vaccine uptake, it was not possible to identify patients who were invited for vaccination though based on age alone, 86% of cases were eligible. Approximately 70% took the vaccine at least once during the 1-3 years period before diagnosis; 15% did not attend for their vaccination and attendance for the remaining 18% was unknown. Patients who attended twice in the 1-3-year period before diagnosis had lower odds of early death than patients who did not attend. Smoking status was not significantly associated with early death with the exception of those who had an unknown smoking status (see Table 1).

Being underweight (BMI <18.5) was strongly associated with early death compared to patients with a normal or elevated BMI. However, obesity was not associated with early death when compared with being non-obese (Table 2). Co-morbidity was common among patients who died early. Almost three quarters (72%) had at least one co-morbidity. CCI score was, however, not associated with early death when means were compared by way of t-test (mean CCI score for cases was 4.75 compared to 4.90 for controls). Dementia was the only co-morbidity within the CCI that was associated with early death - it was present in 8% of cases compared to 4% of controls (Table 2).

Table 2: Presence of individual co-morbidities included in the Charlson score in cases and controls and associated clogit odds ratios and 95% confidence intervals

Characteristic		Case		Control		OR	95% CI
		n	%	n	%		
Underweight	No	467	96.5	479	99.0	1	
	Yes	17	3.5	5	1.0	3.4	1.25–9.22
Obese	No	425	87.8	420	86.8	1	
	Yes	59	12.2	64	13.2	0.91	0.63–1.33
Dementia	No	445	91.9	462	95.5	1	
	Yes	39	8.1	22	4.6	1.85	1.07–3.19
Hypertension	No	279	57.6	260	53.7	1	

For peer review only - http://bmjopen.bmj.com/site/about/guidelines.xhtml

	Yes	205	42.4	224	46.3	0.84	0.6–1.10
Ischaemic heart disease	No	391	80.8	386	79.8	1	
	Yes	93	19.2	98	20.3	0.94	0.68–1.29
Parkinson's Disease	No	473	97.7	477	98.6	1	
	Yes	11	2.3	7	1.5	1.57	0.61–4.05
Valvular heart disease	No	422	87.2	416	86.0	1	
	Yes	62	12.8	68	14.1	0.89	0.61–1.31
Myocardial Infarction	No	429	88.6	436	90.1	1	
	Yes	55	11.4	48	9.9	1.16	0.78–1.72
Congestive heart failure	No	459	94.8	451	93.2	1	
	Yes	25	5.2	33	6.8	0.72	0.41–1.27
Peripheral vascular disease	No	452	93.4	462	95.5	1	
	Yes	32	6.6	22	4.6	1.53	0.86–2.72
Cerebrovascular disease	No	433	89.5	440	90.9	1	
	Yes	51	10.5	44	9.1	1.18	0.77–1.81
COPD	No	402	83.1	397	82.0	1	
	Yes	82	16.9	87	18.0	0.98	0.64–1.50
Connective tissue disorder	No	441	91.1	440	90.9	1	
	Yes	43	8.9	44	9.1	0.98	0.64–1.50
Diabetes without complications	No	420	86.8	429	88.6	1	
	Yes	64	13.2	55	11.4	1.20	0.81–1.77
Peptic ulcer	No	446	92.2	456	94.2	1	
	Yes	38	7.9	28	5.8	1.40	0.84–2.34
Liver disease	No	481	99.4	481	99.4	1	
	Yes	3	0.6	3	0.6	1	0.20–4.95
Hemiplegia / Paraplegia	No	484	100.0	482	99.6	1	
	Yes	0	0.0	2	0.4	-	-

For peer review only - http://bmjopen.bmj.com/site/about/guidelines.xhtml

Renal disease	No	441	91.1	449	92.8	1	
	Yes	43	8.9	35	7.2	1.24	0.79–1.94
Diabetes with complications	No	465	96.1	460	95.1	1	
	Yes	19	3.9	24	5.0	0.79	0.43–1.45
Cancer	No	468	96.7	456	94.2	1	
	Yes	16	3.3	28	5.8	0.57	0.31–1.06
Leukaemia	No	482	99.6	482	99.6	1	
	Yes	2	0.4	2	0.4	1.00	0.14–7.10
Lymphoma	No	482	99.6	483	99.8	1	
	Yes	2	0.4	1	0.2	2.00	0.18–22.06
Severe liver disease	No	484	100	483	99.8	1	
	Yes	0	0	1	0.2	-	-
Metastatic cancer	No	451	93.2	436	90.0	1	
	Yes	33	6.8	48	9.9	0.64	0.40–1.04

† Conditional logistic regression odds ratios for cases and controls matched on age, sex and year diagnosis

Regarding pre-existing psychiatric conditions, 1% of cases were recorded as having schizophrenia, 1% with a learning disability and 13% with anxiety or depression. None were significantly associated with early death. However, the small number of patients with 'other' psychiatric conditions had higher odds of early death (Table 3). Compared to Dukes Stage A, Dukes Stage D and unknown stage were associated with early death. Unknown histological type, unspecified anatomical site and undetermined grade were also associated with early death. Patients with a family history of colorectal cancer had lower odds of early death compared to patients without a family history (Table 4).

For peer review only - http://bmjopen.bmj.com/site/about/guidelines.xhtml

Table 3: Psychiatric illness among cases and controls and associated clogit odds ratios and 95% confidence intervals

Characteristic		Case n	Case %	Control n	Control %	OR	95% CI
Learning disability	No	479	99.0	482	99.6	1	
	Yes	5	1.0	2	0.4	1.24	0.85–1.79
Anxiety/Depression	No	419	86.6	425	87.8	1	
	Yes	65	13.4	59	12.2	1.13	0.76–1.70
Schizophrenia	No	480	99.2	479	99.0	1	
	Yes	4	0.8	5	1.0	0.80	0.22–2.98
Other psychiatric disorder	No	470	97.1	480	99.2	1	
	Yes	14	2.9	4	0.8	3.50	1.15–10.63

† Conditional logistic regression odds ratios for cases and controls matched on age, sex and year diagnosis

Table 4: Disease characteristics among cases and controls and associated odds ratios and 95% confidence intervals

Characteristic		Case n	Case %	Control n	Control %	OR†	95% CI
Anatomical Location	Ascending	52	10.7	57	11.8	1	
	Caecum	93	19.2	136	28.1	0.83	0.52–1.29
	Other	90	18.6	93	19.2	1.19	0.73-1.93
	Descending	29	6.0	18	3.7	1.97	0.97–3.99
	Sigmoid colon	95	19.6	132	27.3	0.86	0.54–1.37
	Not specified	125	25.8	48	9.9	2.96	1.75-5.02
Histological type	Adenocarcinoma	256	52.9	384	79.3	1	
	Mucinous	14	2.9	28	5.8	0.91	0.45–1.81
	Not specified	198	40.9	60	12.4	5.62	3.80–8.34

	Other	16	3.3	12	2.5	1.68	0.77–3.67
Metastases	None	37	7.6	64	13.2	1	
	Bone	9	1.9	1	0.2	17.2	2.48-143.6
	Liver	134	27.7	108	22.3	2.37	1.42-3.95
	Lung	14	2.9	10	2.1	2.55	1.01-6.38
	Other	27	5.6	23	4.8	2.17	1.07-4.41
	Unknown	263	54.3	278	57.4	1.77	1.10-2.84
Dukes Stage	A	8	1.7	19	3.9	1	
	B	46	9.5	85	17.6	1.37	0.54–3.48
	C	47	9.7	127	26.2	1.02	0.41–2.52
	D	160	33.1	140	28.9	2.96	1.23–7.14
	Unknown	233	46.1	113	23.4	5.65	2.30–13.91
Grade (differentiation)	Well/Moderate	93	19.2	190	39.3	1	
	Poor/ Undifferentiated	40	8.3	57	11.8	1.45	0.89-2.36
	Not determined	351	72.5	237	50.0	3.32	2.38-4.62
Colorectal polyps	No	467	10.7	57	11.8	1	
	Yes	17	19.2	136	28.1	0.80	0.42–1.54
Bowel cancer family history	No	459	25.8	48	9.9	1	
	Yes	25	6.0	18	3.7	0.52	0.31–0.88

† Conditional logistic regression odds ratios for cases and controls matched on age, sex and year diagnosis

Multivariable analysis

Unknown marital status, being single, widowed, divorced and living alone were all associated with early death compared to patients who were married/co-habiting after adjusting for other patient characteristics and co-morbidities. Regarding socio-economic status, a deprivation

For peer review only - http://bmjopen.bmj.com/site/about/guidelines.xhtml

gradient for early death was apparent in older people living within quintile 5. This relationship existed for all patients in quintile 4. These socially deprived groups had higher odds of early death compared to the least deprived after adjusting for other factors (Table 5). Being under-weight between 1-3 years before diagnosis was significantly associated with early death in multivariable analysis. Unspecified histology and Dukes Stage D disease remained positively associated with early death in multivariable analysis. Additional models that adjusted for pathway characteristics (attendance at A&E and number of GP consultations prior to diagnosis) did not explain the association between marital status and early death ($p<0.01$). Flu vaccination attendance and baseline consultation activity, dementia, psychiatric illness and smoking status were not significantly associated with early death in multivariable analysis.

Table 5: Multivariable analysis of the association between patient characteristics and early death

		All patients			Male			Female			Aged ≥78 year		
		n≠	OR	95% CI	n≠	OR	95% CI	n≠	OR	95% CI	n≠α	OR	95% CI
Marital / accommodation status	Married and co-habiting	371	1.00		248	1.00		123	1.00		125	1.00	
	Married not co-habiting	26	1.90	0.65-5.57	11	1.27	0.27-6.01	15	3.72	0.70-19.7	17	1.60	0.45-5.67
	Institution care	63	1.93	0.93-4.00	25	1.78	0.57-5.57	38	2.60	0.85-7.82	52	3.02	1.22-7.49
	Unknown	127	5.01	2.82-8.89	66	3.87	1.76-8.38	61	8.49	3.12-23.1	68	4.32	1.97-9.47
	Living alone & SWD*	270	1.64	1.07-2.50	113	1.55	0.86-2.80	157	2.21	1.01-4.80	168	1.62	0.90-2.91
	Other & SWD*	111	1.39	0.80-2.44	47	1.28	0.59-2.74	64	2.03	0.76-5.44	61	0.85	0.37-1.95

For peer review only - http://bmjopen.bmj.com/site/about/guidelines.xhtml

**Deprivation quintile**	Q1 Least deprived	153	1.00		87	1.00		66	1.00		83	1.00	
	Q2	187	1.28	0.74-2.25	91	0.84	0.39-1.82	96	2.90	1.10-7.6	96	2.29	1.06-4.93
	Q3	217	1.42	0.83-2.42	112	1.28	0.62-2.63	105	2.09	0.81-5.4	107	2.05	0.96-4.38
	Q4	212	1.88	1.09-3.23	112	1.60	0.79-3.22	100	3.10	1.14-8.4	113	2.02	0.98-4.15
	Q5 Most deprived	199	1.72	0.98-3.02	108	1.13	0.53-2.33	91	4.70	1.68-13.2	92	2.64	1.15-6.06
**Being underweight (BMI <18.5)**		22	3.99	1.14-14.0	6	2.74	0.32-21.1	16	3.96	0.65-24.2	13	7.27	1.42-68.8
**Dukes stage**	A	8	1.00		20	1.00		7	1.00		10	1.00	
	B	46	1.85	0.64-5.32	70	1.72	0.52-5.67	61	4.90	0.32-75.1	69	2.43	0.43-13.7
	C	47	1.51	0.54-4.27	82	1.13	0.33-3.82	92	6.04	0.43-83.9	88	2.17	0.41-11.6
	D	160	3.07	1.13-8.38	162	1.98	0.63-6.21	138	13.8	1.02-188	109	4.64	0.84-25.7
	Unknown	233	2.99	1.05-8.53	176	1.86	0.58-6.02	160	15.8	1.06-236	215	4.27	0.79-22.9
**Histology type**	Adeno-carcinoma	440	1.00		352	1.00		288	1.00		296	1.00	
	Mucinous	42	1.20	0.54-2.66	17	2.13	0.59-7.64	25	0.87	0.27-2.72	22	1.06	0.35-3.27
	Not specified	258	3.66	2.17-6.17	126	5.02	2.47-10.2	132	2.01	0.82-4.90	166	2.70	1.37-5.31
	Other	28	1.16	0.48-2.83	15	1.58	0.44-5.66	13	0.76	0.17-3.46	7	0.58	0.09-3.77
**Rural residence**		344	1.40	0.98-2.01	182	1.29	0.78-2.17	162	1.53	0.86-2.74	178	1.96	1.21-3.17

**=SWD= single, widowed, divorced*

≠: n= cases and controls combined

For peer review only - http://bmjopen.bmj.com/site/about/guidelines.xhtml

Consistent patterns were observed among males and females when data was stratified by sex though the association between deprivation and early death was strongly pronounced among women and not present among men. Age stratification showed the odds of early death were higher for those living in rural areas compared to urban areas among patients aged ≥78 years. This association was not apparent in those aged <78. Otherwise, there were no significant differences in the factors associated with early death between those aged <78 and those aged ≥78 (see Table 5 – only data for ≥78 shown).

For peer review only

For peer review only - http://bmjopen.bmj.com/site/about/guidelines.xhtml

DISCUSSION

Summary of findings

This study investigated characteristics of patients who died within three months of a diagnosis of colon cancer, by way of univariate and multivariate analyses, with the aim of identifying specific patient factors that can be addressed or accounted for to improve survival outcomes. Social isolation was identified as a common characteristic of early death in colon cancer patients. The different forms of social isolation studied included living alone and being unmarried (as opposed to co-habiting with a partner), residing in more deprived communities (as opposed to living in quintile 1-2 communities), living in a rural area when elderly (as opposed to an urban area) and having dementia or a psychiatric illness. Each of these factors was comparatively associated with early death. Previous studies suggested that poorer outcomes for unmarried people and those living alone were mediated through weak social support[28], that exerted its influence on outcomes through later presentation[29]. This view is consistent with several other studies that reported more negative cancer beliefs[30], lower symptom awareness and greater perceived barriers to GP help-seeking among this group[31]. While weaker social support has previously been associated with later stage disease[32- 33], in this study, despite the collection of a range of detailed pathway and treatment variables, the association between marital status, accommodation status and early death remained unexplained. Other studies suggested biopsychosocial explanations for poorer cancer outcomes in unmarried cancer patients, including chronic stress[34] and weaker immune response[35]. Marital status and accommodation status have an important association with cancer and health outcomes generally[36], yet it is a research area that remains relatively under-investigated.

As in other studies, deprivation quintile was associated with early death, with a gradient in the odds of early death with increasing deprivation score[4,37-38]. However, unlike other studies this association seems restricted to women. Like marital / accommodation status, there was little evidence from this study suggesting that the association between deprivation and early death

For peer review only - http://bmjopen.bmj.com/site/about/guidelines.xhtml

is explained by characteristics of the pathway to diagnosis as the associations persisted after adjusting for attendance at A&E in the pathway to diagnosis, Dukes Stage and GP episodes in the three months to diagnosis. There is no direct biological basis for an association between deprivation and survival. Mediating factors may include lower performance status due to higher tobacco consumption[39], lower uptake of treatments due to fatalistic cancer beliefs[40] or differential access to services[41]. The association between rural residence and early death among the oldest patients was independent of deprivation and strongly significant. This is likely to relate to either isolation, access to services or both. This is a well-defined target group for early diagnosis interventions, but further study is required to investigate the link between lack of social contact and cancer survival if one exists.

Similar to previous work on early colorectal cancer death[4], a consistent feature associated with early death was that of incomplete data due to limited diagnostic testing. Missing histology, stage, grade and anatomical site may be explained by very ill patient's not receiving complete investigation. These characteristics may therefore be viewed as confounding by indication; as opposed to explaining early death, the associations are explained by early death. The relationships between unknown marital, accommodation and outcomes are more difficult to explain. Despite the fact that GP records and secondary care databases were searched, and the information relates to a period over a year prior to diagnosis of cancer, a strong association with early death was observed indicating the data was not missing at random. Missing data on living status, smoking status and alcohol consumption were also associated with early death in univariate analysis. Further work is required to explain this relationship with possible areas for investigation including the patient/practitioner relationship. While co-morbidity was a common feature of patients in this study, we did not find it associated with dying within three months of diagnosis. Previous studies have observed that co-morbidity exerted the greatest influence in the later phases of the survival pathway[42] though CCI score has been observed as an independent indicator of early death elsewhere[4]. We had matched cases and controls on age and as co-morbidity is strongly linked to age, this may have reduced

For peer review only - http://bmjopen.bmj.com/site/about/guidelines.xhtml

For peer review only

our ability to detect this as an independent factor. In addition, being underweight had a strong independent association with early death. This was measured between one and three years before diagnosis and it is likely either related to disease progressing over a longer time period or to poorer performance status. However, as only 17 cases were described as underweight it explains less than 4% of the total early deaths.

While the CCI was not associated with early death, dementia and other psychiatric illness, as individual co-morbid conditions, were associated with early death. However, in multivariable analysis these relationships did not persist. Those with dementia were more likely to have missing data on stage and anatomical location, perhaps suggesting that these patients were less likely to be investigated for their disease. Similar findings have been reported in other colon cancer studies, with dementia associated with poorer colorectal cancer outcomes, later stage disease[43,] and less invasive investigation[44]. The relationship between other psychiatric illness and early death was also attenuated by stage again suggesting a role of diagnostic intervals on the pathway to diagnosis explaining early death. Underlying causes of delay may relate to symptom recognition by carer, patient, practitioner as well as patient communication or competing health care priorities. A key objective of this study was to determine if patient health-seeking characteristics were associated with early death following colon cancer diagnosis. Uptake of the flu vaccine, baseline consultation activity and non-attendance at appointments were identified as three easily captured indicators of health-seeking behaviour. It was hypothesised that patients with more regular or more compliant health-seeking behaviour would have better outcomes than those without, mediated through longer diagnostic intervals and later stage disease.

With regard to health seeking behaviour, although attendance for the flu vaccine was inversely associated with early death, this association was not significant in multivariable analysis. While attendance for the vaccine may be considered an indicator of health compliance, it is likely to reflect various health attitudes and behaviours, with previous UK studies reporting several

For peer review only - http://bmjopen.bmj.com/site/about/guidelines.xhtml

social and cultural factors that were associated with flu vaccination uptake as well as perception regarding health status and susceptibility to flu[45]. The other indicator of health seeking, frequent GP attendance, was correlated with co-morbidity in this study; a relationship reported in several other research studies[10,46]. Although ex-smokers were identified in univariate analysis as having non-significantly lower odds of early death, this association was largely explained in the minimally adjusted model with little evidence to suggest that poorer outcomes among smokers is real. While previous studies have demonstrated a significant association between alcohol-related hospital admissions and early death following a colorectal cancer diagnosis[38], this association was not observed in the current study possibly because this study was unable to discriminate between heavy and moderate alcohol use.

Strengths and weaknesses

The study sample was generated from a high-quality population-based cancer registry system with relatively few DCO cases[47] with full access to General Practice records and hospital clinical records. While missing data was a feature of the study, rather than acting as an impediment to our understanding of characteristics of early death, this appears to be one of the defining features of this patient group. Another recent study of early death has reported a similar pattern of high levels of missing data in those with the poorest outcomes[4]. However, the fact that remains that missing data was a feature of this study and potential improvements to the way data is recorded in the NICR are continuously being made. The study investigated a broad range of factors that may be associated with early death and allowed for adjustment of a range of confounding factors such as co-morbidity, smoking and alcohol status. We present results from both univariate and multivariate analysis but place greater emphasis upon results from multivariate analysis due to the complex nature of interacting factors causing early death from colon cancer. The recording of the patient characteristics that vary over time at between one and three years before diagnosis was an important feature of the study design. Cancer diagnosis has been previously identified as being associated with changes in health-seeking behaviour, co-morbidity, BMI and lifestyle factors. Recording these factors based on

For peer review only - http://bmjopen.bmj.com/site/about/guidelines.xhtml

over one year before diagnosis strengthens the assessment of causal inference between variables and early death.

The case-control design in the study provided an efficient methodology to collect data and allowed the development of a control group that was matched on important but non-modifiable characteristics. While age, sex and year of diagnosis were fixed in the current study, their interaction with diagnostic pathway features and other characteristics could not be investigated. Previous studies have shown longer diagnostic timelines, later stage disease at diagnosis, lower symptom awareness and more negative cancer beliefs to be variable depending on age[48] and sex[49]. In addition, the matching on age may have reduced variation in other characteristics such as co-morbidity. The study population was also selected in a period before the introduction of screening, therefore all patients in both the case and control group were clinically diagnosed. While bowel screening was introduced in NI in 2011[23] and now represents an important pathway in cancer diagnosis, the majority of patients are still diagnosed clinically and the early clinical detection of symptoms remains both important and relevant – particularly for cancer stage and by extension, cancer survival. Future iterations of this work would likely benefit from matching of cases and controls on cancer stage in addition to age, sex, and year of diagnosis.

The use of a control group of longer survivors provided useful comparative information to investigate risk of early death, with the choice of a control sample of deceased colon cancer patients removing any risk of consent bias from the study. However, while a buffer of three months was placed between cases and controls to allow better discrimination between the two, the survival of controls was restricted to a population of patients whose survival was less than three years and similar to the case population. Despite this, the study was able to identify several characteristics which discriminated between cases and controls suggesting that the patients who die within the first few months of diagnosis are a specific patient cohort who require attention.

For peer review only - http://bmjopen.bmj.com/site/about/guidelines.xhtml

For peer review only

CONCLUSIONS

This comprehensive study of early death from colon cancer has identified several population sub-groups that warrant special attention. These include those who are single, living alone, older people living in rural environments, and people from the most deprived communities as well as those living in residential or nursing care. These likely comprise some of the most isolated people in society. However, while the aforementioned variables are an indicator of social isolation, this study was not designed to actually *investigate* isolation (i.e. lack of social contact or poor social support networks). Therefore, further study is required to confirm that social isolation is definitely linked with poor cancer survival outcomes. Further studies are also required to better understand the role of missing data in patient records. Furthermore, additional work ought to be undertaken to determine if these patterns are consistent in other ICBP countries. Finally, because of increased colon cancer survival, future studies investigating risk factors for an *early death* using a case control methodology would likely benefit from comparing cases who suffer early mortality with controls who survive beyond five or perhaps even ten years as opposed to the three-year survival control group used in this study[50-52].

ACKNOWLEDGEMENTS

The authors gratefully appreciate the contribution of data abstractors: Donna Floyd, Rosemary Ward, Jacqui Napier, Kate Donnelly and Bríd Morris. The authors also thank Business Services Organisation, Health & Social Care NI for facilitating the note review.

FUNDING

The TEDI study was funded by the Cancer Research UK led National Awareness & Early Diagnosis Initiative. The Northern Ireland Cancer Registry is funded by the Public Health Agency Northern Ireland.

For peer review only - http://bmjopen.bmj.com/site/about/guidelines.xhtml

DATA SHARING

Data from the TEDI study is available from the N. Ireland Cancer Registry with approval from the data custodian. Interested parties should contact Dr Anna Gavin, Director, N. Ireland Cancer Registry.

COMPETING INTERESTS

There are no competing interests to be reported by the authors in this study

CONTRIBUTOR STATEMENT

CD designed the study, undertook all statistical analysis and drafted the manuscript. NH was medical adviser for the study, provided advice on statistical analysis and interpretation and contributed to the writing of the manuscript. AM contributed to the writing, editing and submission of the paper. MD provided advice and guidance on the study design and contributed to writing of the manuscript. LA provided advice and guidance on the study design and contributed to writing of the manuscript. LR contributed to the study design, development of data collection forms and data abstractor training and analysis and interpretation of results. AG was Principal Investigator, funding recipient and contributed to the writing of the manuscript.

For peer review only

REFERENCES

1 Coleman MP, Forman D, Bryant H, *et al*. Cancer survival in Australia, Canada, Denmark, Norway, Sweden, and the UK, 1995-2007 (the International Cancer Benchmarking Partnership): an analysis of population-based cancer registry data. *Lancet*. 2011; 377(9760): 127-138.

2 De Angelis R, Sant M, Coleman MP, *et al*. Cancer survival in Europe 1999–2007 by country and age: results of EUROCARE-5—a population-based study. *Lancet Oncol*. 2014; 15(1): 23-34.

3 Morris EJ, Sandin F, Lambert PC, *et al*. A population-based comparison of the survival of patients with colorectal cancer in England, Norway and Sweden between 1996 and 2004. *Gut*. 2011; 60: 1087-1093.

4 Downing A, Aravani A, Macleod U, *et al*. Early mortality from colorectal cancer in England: a retrospective observational study of the factors associated with death in the first year after diagnosis. *Br J Cancer*. 2013; 108(3): 681-685.

5 Northern Ireland Cancer Registry. Official Statistics on cancer incidence and survival: All Cancers (Excluding Non-Melanoma Skin Cancer) 2015. http://www.qub.ac.uk/researchcentres/nicr/FileStore/PDF/FactSheets/Filetoupload,531393,en.pdf (accessed 15 March 2017).

6 Thomson CS, Forman D. Cancer survival in England and the influence of early diagnosis: what can we learn from recent EUROCARE results?. *Br J Cancer*. 2009; 101(2): S102–S109.

7 Sogaard M, Thomsen RW, Bossen KS, *et al*. The impact of comorbidity on cancer survival: a review. *Clin Epidemiol*. 2013; 5(1): 3-29.

8 Genevieve M, Boland GJ, Chang AB, *et al*. Association between Adherence to National Comprehensive Cancer Network Treatment Guidelines and Improved Survival in Patients with Colon Cancer. *Cancer*. 2013; 119(8): 1593–1601.

For peer review only - http://bmjopen.bmj.com/site/about/guidelines.xhtml

9 Mitchell E, Macdonald S, Campbell NC, *et al*. Influences on pre-hospital delay in the diagnosis of colorectal cancer: a systematic review. *Br J Cancer*. 2008; 98(1): 60-70.

10 Marshall JR, Funch DP. Gender and illness behaviour among colorectal cancer patients. *Women and Health*. 1986; 11: 67–82.

11 Mariscal M, Llorca J, Prieto D, *et al*. Determinants of the interval between the onset of symptoms and diagnosis in patients with digestive tract cancers. *Cancer Detect Prevent*. 2001; 25(5): 420–429.

12 McCaffery K, Wardle J, Waller J. Knowledge, attitudes, and behavioural intentions in relation to the early detection of colorectal cancer in the United Kingdom. *Prev Med*. 2003; 36: 525–535.

13 Robinson E, Mohilever J, Zidan J, *et al*. Colorectal cancer: incidence, delay in diagnosis and stage of disease. *Eur J Cancer Clin Oncol*. 1986; 22(2): 157–161.

14 Bain NSC, Campbell NC, Ritchie LD, *et al*. Striking the right balance in colorectal cancer – a qualitative study of rural and urban patients. *Fam Pract*. 2002; 19(4): 369–374.

15 Holliday HW, Hardcastle JD. Delay in diagnosis and treatment of symptomatic colorectal cancer. *Lancet*. 1979; 1: 309–311.

16 MacAdam DB. A study in general practice of the symptoms and delay patterns in the diagnosis of gastrointestinal cancer. *J R Coll Gen Pract* 1979; 29: 723–729.

17 Mor V, Masterson-Allen S, Goldberg R, *et al*. Prediagnostic symptom recognition and help-seeking among cancer patients. *J Community Health*. 1990; 15(4): 253–266.

18 Curless R, French JM, Williams GV, *et al*. Colorectal carcinoma: do elderly patients present differently? *Age Ageing*. 1994; 23: 102–107.

19 Mitchell E, Macdonald S, Campbell NC, *et al*. Influences on pre-hospital delay in the diagnosis of colorectal cancer: a systematic review. *Br J Cancer*. 2008; 98: 60–70.

20 Nilsson E, Bolin S, Sjodahl R. Carcinoma of the colon and rectum. Delay in diagnosis. *Acta Chir Scand*. 1982; 148: 617.

21 Roncoroni L, Pietra N, Violi V, *et al*. Delay in the diagnosis and outcome of colorectal cancer: a prospective study. *Eur J Surg Oncol*. 1999; 25: 173–178.

For peer review only - http://bmjopen.bmj.com/site/about/guidelines.xhtml

22 Weller D, Coleman D, Robertson R, *et al*. The UK colorectal cancer screening pilot: results of the second round of screening in England. *Br J Cancer*, 2007; *97*(12): 1601-1605.

23 Bradley DT, Treanor C, McMullan C, *et al*. Reasons for non-participation in the Northern Ireland Bowel Cancer Screening Programme: a qualitative study. *BMJ Open*. 2015; 5: e008266.

24 Elliss-Brookes L, McPhail S, Ives A, *et al*. Routes to diagnosis for cancer–determining the patient journey using multiple routine data sets. *Br J Cancer*. 2012; 107(8): 1220-1226.

25 Weller D, Vedsted P, Rubin G, *et al*. The Aarhus statement: improving design and reporting of studies on early cancer diagnosis. *Br J Cancer*. 2012; 106(7): 1262-1267.

26 Lunt M. Optimal matching for case control studies, Computer program and documentation for use with Stata statistical software. Available at http://personalpages.manchester.ac.uk/staff/mark.lunt/optmatch2.hlp; 2007 (accessed 24 December 2013).

27 Rosenbaum PR. Optimal matching for observational studies. *J Am Stat Assoc*. 1989; 1024–1032.

28 Villingshøj M, Ross L, Thomsen BL, *et al*. Does marital status and altered contact with the social network predict colorectal cancer survival? *Eur J Cancer.* 2006; 42(17):3022-3027.

29 Mitchell ED, Pickwell-Smith B, Macleod U. Risk factors for emergency presentation with lung and colorectal cancers: a systematic review. *BMJ Open*. 2015; 5(4): e006965.

30 Quaife SL, Winstanley K, Robb KA, *et al*. Socioeconomic inequalities in attitudes towards cancer: an international cancer benchmarking partnership study. Eur J Can Prev. 2015; 24(3): 253.

31 Niksic M, Rachet B, Warburton FG, *et al*. Cancer symptom awareness and barriers to symptomatic presentation in England—are we clear on cancer?. Br J Can. 2015; 113(3): 533-542.

For peer review only - http://bmjopen.bmj.com/site/about/guidelines.xhtml

32 Aizer AA, Chen MH, McCarthy EP, *et al*. Marital status and survival in patients with cancer. *J Clin Oncol*. 2013; 31: 3869–3876.

33 Nayeri K, Pitaro G, Feldman JG. Marital status and stage at diagnosis in cancer. *N Y State J Med*. 1992; 92: 8–11.

34 Levy SM, Herberman RB, Whiteside T, *et al*. Perceived social support and tumour estrogen/progesterone receptor status as predictors of natural killer cell activity in breast cancer patients. *Psychosomatic Medicine*. 1990; 52: 73–85.

35 Hamilton TD, Leugner D, Kopciuk K, *et al*. Identification of prognostic inflammatory factors in colorectal liver metastases. *BMC Cancer*. 2014; 14: 542.

36 Berkman LF. The role of social relations in health promotion. *Psychosomatic Medicine*. 1995; 57(3): 245-254.

37 Henrik Møller, Fredrik Sandin, David Robinson, *et al*. Colorectal cancer survival in socioeconomic groups in England: Variation is mainly in the short term after diagnosis. *Eur J Cancer*. 2012; 48(1): 46–53.

38 Brewster DH, Clark DI, Stockton DL, *et al*. Characteristics of patients dying within 30 days of diagnosis of breast or colorectal cancer in Scotland, 2003–2007. *Br J Cancer*. 2011; 104(1): 60–67.

39 Munro AJ, Bentley AHM, Ackland C, *et al*. Smoking compromises cause-specific survival in patients with operable colorectal cancer. *Clin Oncol*. 2006; 18(6): 436-440.

40 Beeken RJ, Simon AE, Von Wagner C, *et al*. Cancer fatalism: deterring early presentation and increasing social inequalities? *Cancer Epidemiology Biomarkers & Prevention* 2011; *20*(10): 2127-2131.

41 Raine R, Wong W, Scholes S, *et al*. Social variations in access to hospital care for patients with colorectal, breast, and lung cancer between 1999 and 2006: retrospective analysis of hospital episode statistics. *BMJ*. 2010; *340*: b5479.

42 Sogaard M, Thomsen RW, Bossen KS, *et al*. The impact of comorbidity on cancer survival: a review. *Clin Epidemiol*. 2013; 5(1): 3-29.

For peer review only

43 Gupta SK, Lamont EB. Patterns of presentation, diagnosis, and treatment in older patients with colon cancer and comorbid dementia. *J Am Ger Soc*. 2004; 52: 1681-1687.

44 Raji MA, Kuo YF, Freeman JL, *et al*. Effect of a dementia diagnosis on survival of older patients after a diagnosis of breast, colon, or prostate cancer: implications for cancer care. Arch Intern Med. 2008; 168(18): 2033-2040.

45 Nagata JM, Hernández-Ramos I, Kurup AS, *et al*. Social determinants of health and seasonal influenza vaccination in adults≥ 65 years: a systematic review of qualitative and quantitative data. *BMC Public Health*. 2013; 13(1): 1.

46 Van Oostrom SH, Picavet HSJ, De Bruin SR, *et al*. Multimorbidity of chronic diseases and health care utilization in general practice. *BMC Fam Pract*. 2013; 15(1): 1.

47 Kearney TM, Donnelly C, Kelly JM, *et al*. Validation of the completeness and accuracy of the Northern Ireland Cancer Registry. *Cancer Epidemiology*. 2013; 39(3): 401-404.

48 Forbes LJ, Simon AE, Warburton F, *et al*. Differences in cancer awareness and beliefs between Australia, Canada, Denmark, Norway, Sweden and the UK(the International Cancer Benchmarking Partnership): do they contribute to differences in cancer survival? *Br J Cancer*. 2013; 108(2): 292-300.

49 Evans REC, Brotherstone H, Miles A, *et al*. Gender differences in early detection of cancer. *J Men's Health Gender*. 2005; 2: 209–217.

50 Araghi M, Soerjomataram I, Jenkins M, *et al*. Global trends in colorectal cancer mortality: projections to the year 2035. *Int J Cancer*. 2019 [ePub ahead of print]. doi: 10.1002/ijc.32055.

51 Colon cancer mortality statistics 1993-2017. N. Ireland Cancer Registry. https://www.qub.ac.uk/research-centres/nicr/FileStore/OfficialStats2017/Mortality/Filetoupload,883604,en.xls (accessed 19 March 2019).

52 Bowel cancer survival statistics. Cancer Research UK. https://www.cancerresearchuk.org/sites/default/files/cstream-node/surv_1_5_10yr_bowel.pdf (accessed 19 March 2019).

For peer review only - http://bmjopen.bmj.com/site/about/guidelines.xhtml

Figure legend

Figure 1: case inclusions and exclusions

For peer review only

For peer review only - http://bmjopen.bmj.com/site/about/guidelines.xhtml

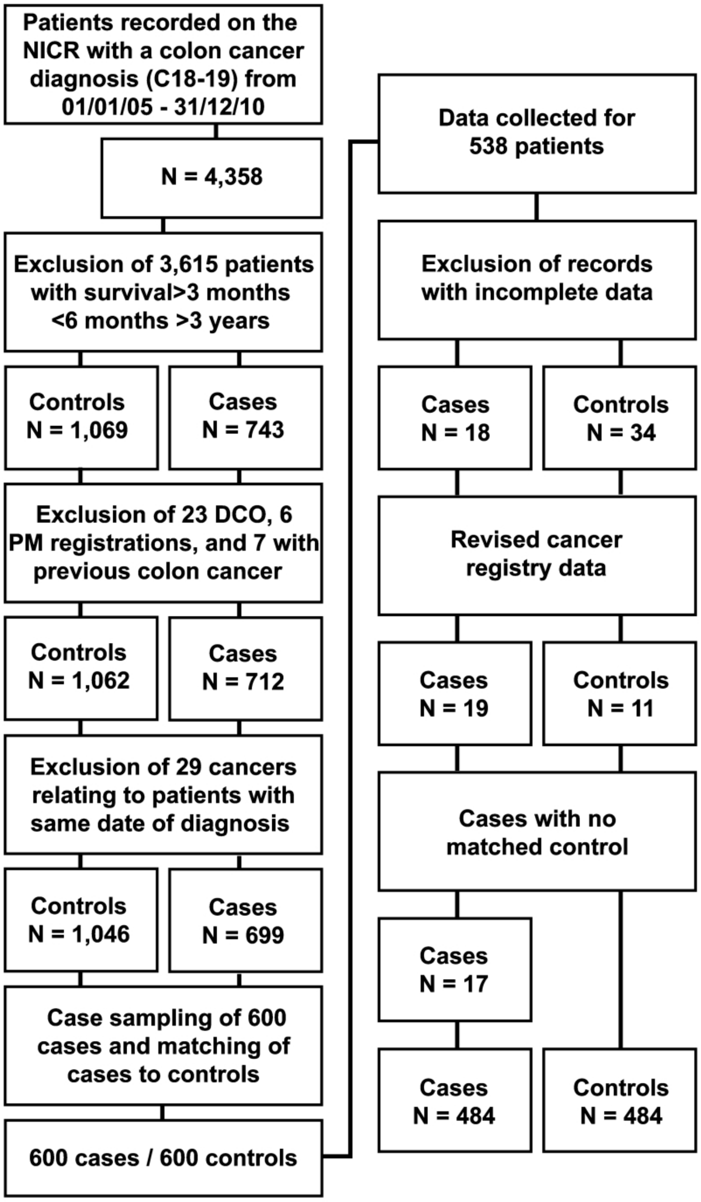

Case inclusions and exclusions

144x247mm (600 x 600 DPI)

Supplementary Table S1: Distribution of the case sample and the remaining patients who died within three months of diagnosis who were not included in the study

Characteristics		Case Sample n=484	Cases not included n=196	P value
Sex %	Male	52.7	44.3	
	Female	47.3	55.7	0.05^{χ}
Year diagnosed %	2005-06	39.2	29.4	
	2007-08	38.5	18.40	
	2009-10	22.3	51.3	$<0.01^{\chi}$
Dukes stage %	A	2.3	1.0	
	B	10.1	7.0	
	C	10.7	9.0	
	D	37.0	44.3	
	Unknown	39.9	38.8	0.27^{χ}
Deprivation quintile %	1 (least deprived)	16.6	15.2	
	2	20.9	22.7	
	3	17.4	18.7	
	4	24.8	21.7	
	5 (most deprived)	20.3	21.7	0.87^{χ}
Mean age at diagnosis in years (Standard deviation)		76.5 (10.6)	77.6 (10.3)	$0.83^{\dagger}$
Mean survival duration in days (Standard deviation)		36.7 (24.7)	33.8 (39.9)	0.82^{β}

P-values presented for chi squared tests (χ), t-tests (†) and Kaplan Meier (β)

For peer review only

STROBE checklist of items to be included in reports of observational studies in epidemiology*
Checklist for cohort, case-control, and cross-sectional studies (combined)

Section/Topic	Item #	Recommendation	Reported on page #
**Title and abstract**	1	(*a*) Indicate the study's design with a commonly used term in the title or the abstract	1
		(*b*) Provide in the abstract an informative and balanced summary of what was done and what was found	2
**Introduction**			
Background/rationale	2	Explain the scientific background and rationale for the investigation being reported	4
Objectives	3	State specific objectives, including any pre-specified hypotheses	5
**Methods**			
Study design	4	Present key elements of study design early in the paper	2 and 5
Setting	5	Describe the setting, locations, and relevant dates, including periods of recruitment, exposure, follow-up, and data collection	2 and 5
Participants	6	(*a*) *Cohort study*—Give the eligibility criteria, and the sources and methods of selection of participants. Describe methods of follow-up<br>*Case-control study*—Give the eligibility criteria, and the sources and methods of case ascertainment and control selection. Give the rationale for the choice of cases and controls<br>*Cross-sectional study*—Give the eligibility criteria, and the sources and methods of selection of participants	5
		(*b*) *Cohort study*—For matched studies, give matching criteria and number of exposed and unexposed<br>*Case-control study*—For matched studies, give matching criteria and the number of controls per case	5
Variables	7	Clearly define all outcomes, exposures, predictors, potential confounders, and effect modifiers. Give diagnostic criteria, if applicable	6
Data sources/ measurement	8*	For each variable of interest, give sources of data and details of methods of assessment (measurement). Describe comparability of assessment methods if there is more than one group	*6*
Bias	9	Describe any efforts to address potential sources of bias	6-7
Study size	10	Explain how the study size was arrived at	6-7
Quantitative variables	11	Explain how quantitative variables were handled in the analyses. If applicable, describe which groupings were chosen and why	6-7
Statistical methods	12	(*a*) Describe all statistical methods, including those used to control for confounding	6-7
		(*b*) Describe any methods used to examine subgroups and interactions	7
		(*c*) Explain how missing data were addressed	Fig 1
		(*d*) *Cohort study*—If applicable, explain how loss to follow-up was addressed<br>*Case-control study*—If applicable, explain how matching of cases and controls was addressed	5

For peer review only

		Cross-sectional study—If applicable, describe analytical methods taking account of sampling strategy	
		(*e*) Describe any sensitivity analyses	-
**Results**			
Participants	13*	(a) Report numbers of individuals at each stage of study—eg numbers potentially eligible, examined for eligibility, confirmed eligible, included in the study, completing follow-up, and analysed	5 and fig 1
		(b) Give reasons for non-participation at each stage	Fig 1
		(c) Consider use of a flow diagram	Fig 1
Descriptive data	14*	(a) Give characteristics of study participants (eg demographic, clinical, social) and information on exposures and potential confounders	Table 1
		(b) Indicate number of participants with missing data for each variable of interest	Table 1-4
		(c) *Cohort study*—Summarise follow-up time (eg, average and total amount)	
Outcome data	15*	*Cohort study*—Report numbers of outcome events or summary measures over time	
		Case-control study—Report numbers in each exposure category, or summary measures of exposure	Table 1-4
		Cross-sectional study—Report numbers of outcome events or summary measures	
Main results	16	(*a*) Give unadjusted estimates and, if applicable, confounder-adjusted estimates and their precision (eg, 95% confidence interval). Make clear which confounders were adjusted for and why they were included	Table 1-5
		(*b*) Report category boundaries when continuous variables were categorized	Table 1-4
		(*c*) If relevant, consider translating estimates of relative risk into absolute risk for a meaningful time period	
Other analyses	17	Report other analyses done—eg analyses of subgroups and interactions, and sensitivity analyses	Table 5
**Discussion**			
Key results	18	Summarise key results with reference to study objectives	18-20
Limitations	19	Discuss limitations of the study, taking into account sources of potential bias or imprecision. Discuss both direction and magnitude of any potential bias	21
Interpretation	20	Give a cautious overall interpretation of results considering objectives, limitations, multiplicity of analyses, results from similar studies, and other relevant evidence	19-20
Generalisability	21	Discuss the generalisability (external validity) of the study results	21-22
**Other information**			
Funding	22	Give the source of funding and the role of the funders for the present study and, if applicable, for the original study on which the present article is based	23

*Give information separately for cases and controls in case-control studies and, if applicable, for exposed and unexposed groups in cohort and cross-sectional studies.
Note: An Explanation and Elaboration article discusses each checklist item and gives methodological background and published examples of transparent reporting. The STROBE checklist is best used in conjunction with this article (freely available on the Web sites of PLoS Medicine at http://www.plosmedicine.org/, Annals of Internal Medicine at http://www.annals.org/, and Epidemiology at http://www.epidem.com/). Information on the STROBE Initiative is available at www.strobe-statement.org.

For peer review only
